# Supplementary material for: Brain MRI and neuropsychological findings at long-term follow-up after COVID-19 hospitalisation: an observational cohort study
Source: BMJ Open. 2021 Oct 27;11(10):e055164. doi: 10.1136/bmjopen-2021-055164 (PMC8551746; doi:10.1136/bmjopen-2021-055164)
Supplement: Supplementary data [file bmjopen-2021-055164supp001.pdf]

Supplemental Table 1. Characteristics for all included patients hospitalised in a Swedish geographical region during the period March 1 to May 31, 2020 with a laboratory confirmed COVID-19; those eligible for interview; those invited for a clinical assessment and those who underwent an MRI

	**All hospitalised (n=734)**	**Eligible for interview (n=460)**	**Invited to a clinical assessment (n=185)**	**MRI performed (n=35)**
Age, median (IQR) years	67 (52-79)	61 (49-74)	59 (49-67)	59 (51-66)
Men/women, n (%)	388 /346 (53/47)	257/203 (56/44)	104/81 (56/44)	28/7 (80/20)
Days in hospital, median (IQR)	6 (3-12)	6 (3-13)	7 (3-19)	18 (7-47)
ICU care, need/no need, n (%)	102/632 (14/86)	72/388 (16/84)	49/136	20/15 (57/43)
Premorbid function category 1/2/3/4/missing, n (%)	210/188/226/100/10 (29/26/31/14/1)	183/142/125/9/1 (40/31/27/2/0)	92/58/32/2/1 (51/31/17/1/1)	20/12/3/0/0 (57/34/9/0/0)
WHO Clinical Progression Scale 4/5/6/7/8/9/10/missing, n (%)	230/261/47/0/25/34/130/7 (31/36/6/0/3/5/18/1)	139/221/41/0/25/34/0/0 (30/48/9/0/5/7/0/0)	51/71/18/0/20/25/0/0 (28/38/10/0/11/14/0/0)	4/8/4/0/6/13/0/0 (11/23/11/0/17/37/0/0)

Categories of premorbid function: 1. No or mild frailty, no restriction in daily life; 2. Moderate frailty, mobile and independent, but unable to handle physically demanding activities or work; 3. Considerable frailty, ability to perform activities of daily living, but in periods confined to bed or chair; and 4. Severe frailty, not able to perform activities of daily living and/or confined to bed or chair. Dementia necessitating care. WHO Clinical Progression Scale: 4. Hospitalised, moderate disease, no oxygen therapy; 5. Hospitalised, moderate disease, oxygen by mask or nasal prongs; 6. Hospitalised, severe diseases, oxygen by non-invasive ventilation or high flow; 7. Hospitalised, severe diseases, intubation and mechanical ventilation, $pO_2/FiO_2 \geq 150$ or $SpO_2/FiO_2 \geq 200$; 8. Hospitalised, severe diseases, mechanical ventilation pO_2/FiO_2 <150 (SpO_2/FiO_2 <200) or vasopressors; and 9. Hospitalised, severe diseases, mechanical ventilation pO_2/FiO_2 <150 and vasopressors, dialysis, or ECMO. Number of symptoms affecting daily life as reported by patients 4 months post discharge.
