# Supplementary material for: Brain MRI and neuropsychological findings at long-term follow-up after COVID-19 hospitalisation: an observational cohort study
Source: BMJ Open. 2021 Oct 27;11(10):e055164. doi: 10.1136/bmjopen-2021-055164 (PMC8551746; doi:10.1136/bmjopen-2021-055164)
Supplement: Supplementary data [file bmjopen-2021-055164supp002.pdf]

Supplemental table 2 MRI findings in patients previously hospitalized with COVID-19

Gender	**Age (years in intervalls)**	**Previous MRI in acute phase**	**SWI abnormalities on MRI**	**White matter lesions on MRI**	**Confluent white matter lesions**	**New white matter lesions**	**Normal MRI**
F	40-49						X
M	40-49						X
M	50-59			X			
F	60-69			X			
M	50-59						X
M	50-59			X	X		
M	40-49						X
M	60-69			X			
M	70-79			X			
F	60-69			X			
M	70-79		X		X		
F	50-59						X
M	60-69	X	X	X	X	X	
M	50-59						X
M	50-59			X			
F	50-59			X			
F	60-69		X				
M	60-69			X	X		
M	50-59			X	X		
M	40-49			X			
M	40-49						X
M	50-59						X
F	60-69						X
M	60-69			X			
M	70-79	X	X	X		X	
M	50-59			X			
M	70-79	X		X	X	X	
M	40-49			X			
M	60-69	X	X	X		X	
M	50-59						X
M	60-69			X			
M	50-59	X		X		X	
M	80-89		X		X		
M	60-69	X	X	X		X	
M	50-59		X				
