# Supplementary material for: Brain MRI and neuropsychological findings at long-term follow-up after COVID-19 hospitalisation: an observational cohort study
Source: BMJ Open. 2021 Oct 27;11(10):e055164. doi: 10.1136/bmjopen-2021-055164 (PMC8551746; doi:10.1136/bmjopen-2021-055164)
Supplement: Supplementary data [file bmjopen-2021-055164supp003.pdf]

Supplemental table 3 Background data and data from the clinical assessment on patients who did/did not complete all RBANS subtests

	Patients who completed all RBANS subtests	Patients who did not complete all RBANS subtests	P [a)]	Patients who completed all RBANS subtests (n)	Patients who did not complete all RBANS subtests (n)	P[b)]	Patients who completed all RBANS subtests (n)	Patients who did not complete all RBANS subtests (n)
Age, median (IQR)[a]	59 (51-66)	62.5 (51.25-76)	n.s.				31	4
Men/women, n[b]				24/7	4/0	n.s.	31	4
Days in hospital, median (IQR)[a]	18 (8-47)	13 (4.75-86.5)	n.s.				31	4
ICU care, need/no need, n[b]				18/13	2/2	n.s.	31	4
Mechanical ventilation, need/no need, n[b]				17/14	2/2	n.s.	31	4
Premorbid function category 1/2/3/4, n[b]				19/10/2/0	1/2/1/0	n.s.	31	4
WHO Clinical Progression Scale 4/5/6/7/8/9, n[b]				3/7/4/0/6/11	1/1/0/0/0/2	n.s.	31	4
Educational level 1/2/3, n[b]				7/15/9	3/0/4	n.s.	31	4
Self-estimated previous health, n[b]				13/9/8/0/0	0/3/1/0/0	n.s.	30	4
Self-estimated present health, n[b]				4/4/16/6/0	0/0/2/2/0	n.s.	30	4
Distribution of impaired cognitive results, category 1/2/3, n[b]				8/6/17	2/0/2	n.s.	31	4
MFI, above cut-off/not above cut-off, n[b]				23/7	3/1	n.s.	30	4
HADS-depression above/not above cut-off, n[b]				3/28	2/2	n.s.	30	3
HADS-anxiety above/not above cut-off, n[b]				4/27	2/2	n.s.	30	3
MRI findings abnormal/normal, n[b]				22/9	3/1	n.s.	31	4

[a)]Median test [b)]Fisher test for comparison of frequencies. The difference was considered significant at $P<0.05$. n.s.=non-significant. n=number of patients. Categories of premorbid function: 1. No or mild frailty, no restriction in daily life; 2. Moderate frailty, mobile and independent, but unable to handle physically demanding activities or work; 3. Considerable frailty, ability to perform activities of daily living, but with periods confined to bed or chair; and 4. Severe frailty, not able to perform activities of daily living and/or confined to bed or chair. Dementia necessitating care. WHO Clinical Progression Scale: 4. Hospitalised, moderate disease, no oxygen therapy; 5. Hospitalised, moderate disease, oxygen by mask or nasal prongs; 6. Hospitalised, severe diseases, oxygen by non-invasive ventilation or high flow; 7. Hospitalised, severe diseases, intubation and mechanical ventilation, $pO_2/FiO_2 \geq 150$ or $SpO_2/FiO_2 \geq 200$; 8. Hospitalised, severe diseases, mechanical ventilation $pO_2/FiO_2 <150$ ($SpO_2/FiO_2 <200$) or vasopressors; and 9. Hospitalised, severe diseases, mechanical ventilation $pO_2/FiO_2 <150$ and vasopressors, dialysis, or extracorporeal membrane oxygenation. Categories of educational level: 1, up to 9 years of school; 2, 9 to 12 years of school; and 3, more than 12 years of school. Categories of self-estimated health (previous and present): 1, Excellent; 2, Very good; 3, Good; 4, Fair; and 5, Poor. RBANS=The Repeatable Battery for Assessment of Neuropsychological Status Categories of impaired cognitive results: 1, scores 2SD below mean according to norms in at least two RBANS indexes, or 1.5SD below the mean according to the norms on at least three RBANS indexes, was considered severe impairment; 2, scores 2SD below the mean according to the norms on one of the RBANS indexes or 1.5SD below the mean according to the norms on two RBANS indexes, was considered mild/moderate impairment; 3, scores not included in categories 1 and 2, considered not mild/moderate/severe impairment. RBANS cut-off (scores at least 2SDs below age-adjusted population mean of 100, SD 15 according to norms). MFI=Multidimensional Fatigue Inventory. MFI cut-off for clinically significant fatigue ≥53. HADS= Hospital Anxiety and Depression Scale. HADS cut-off for potential depression/anxiety ≥8.
