# Supplementary material for: Brain MRI and neuropsychological findings at long-term follow-up after COVID-19 hospitalisation: an observational cohort study
Source: BMJ Open. 2021 Oct 27;11(10):e055164. doi: 10.1136/bmjopen-2021-055164 (PMC8551746; doi:10.1136/bmjopen-2021-055164)
Supplement: Author's manuscript [file bmjopen-2021-055164.draft_revisions.pdf]

BMJ Open

BMJ Open is committed to open peer review. As part of this commitment we make the peer review history of every article we publish publicly available.

When an article is published we post the peer reviewers' comments and the authors' responses online. We also post the versions of the paper that were used during peer review. These are the versions that the peer review comments apply to.

The versions of the paper that follow are the versions that were submitted during the peer review process. They are not the versions of record or the final published versions. They should not be cited or distributed as the published version of this manuscript.

BMJ Open is an open access journal and the full, final, typeset and author-corrected version of record of the manuscript is available on our site with no access controls, subscription charges or pay-per-view fees (http://bmjopen.bmj.com).

If you have any questions on BMJ Open's open peer review process please email info.bmjopen@bmj.com

BMJ Open

Brain MRI and neuropsychological findings at long-term follow-up after COVID-19 hospitalisation: an observational cohort study

Journal:	*BMJ Open*
Manuscript ID	bmjopen-2021-055164
Article Type:	Original research
Date Submitted by the Author:	04-Jul-2021

Complete List of Authors:	Hellgren, Lovisa; Linkoping University Department of Medical and Health Sciences, Department of Rehabilitation Medicine, Region Jönköping County, and Department of Medical and Health Sciences, Linköping University, Linköping, Sweden<br>Birberg Thornberg, Ulrika; Linkoping University Department of Medical and Health Sciences, Department of Rehabilitation Medicine and Department of Medical and Health Sciences, Linköping University, Linköping, Sweden<br>Samuelsson, Kersti; Linkoping University Department of Medical and Health Sciences, Department of Rehabilitation Medicine and Department of Medical and Health Sciences, Linköping University, Linköping, Sweden<br>Levi, Richard; Linkoping University Department of Medical and Health Sciences, Department of Rehabilitation Medicine and Department of Medical and Health Sciences, Linköping University, Linköping, Sweden<br>Divanoglou, Anestis; Linkoping University Department of Medical and Health Sciences, Department of Rehabilitation Medicine and Department of Medical and Health Sciences, Linköping University, Linköping, Sweden<br>Blystad, Ida; Linkoping University Department of Health Medicine and Caring Sciences, Department of Radiology in Linköping and Department of Health, Medicine and Caring Sciences, Linköping University, Linköping, Sweden

Keywords:	COVID-19, Magnetic resonance imaging < RADIOLOGY & IMAGING, REHABILITATION MEDICINE

SCHOLARONE™
Manuscripts

I, the Submitting Author has the right to grant and does grant on behalf of all authors of the Work (as defined in the below author licence), an exclusive licence and/or a non-exclusive licence for contributions from authors who are: i) UK Crown employees; ii) where BMJ has agreed a CC-BY licence shall apply, and/or iii) in accordance with the terms applicable for US Federal Government officers or employees acting as part of their official duties; on a worldwide, perpetual, irrevocable, royalty-free basis to BMJ Publishing Group Ltd ("BMJ") its licensees and where the relevant Journal is co-owned by BMJ to the co-owners of the Journal, to publish the Work in this journal and any other BMJ products and to exploit all rights, as set out in our licence.

The Submitting Author accepts and understands that any supply made under these terms is made by BMJ to the Submitting Author unless you are acting as an employee on behalf of your employer or a postgraduate student of an affiliated institution which is paying any applicable article publishing charge ("APC") for Open Access articles. Where the Submitting Author wishes to make the Work available on an Open Access basis (and intends to pay the relevant APC), the terms of reuse of such Open Access shall be governed by a Creative Commons licence – details of these licences and which Creative Commons licence will apply to this Work are set out in our licence referred to above.

Other than as permitted in any relevant BMJ Author's Self Archiving Policies, I confirm this Work has not been accepted for publication elsewhere, is not being considered for publication elsewhere and does not duplicate material already published. I confirm all authors consent to publication of this Work and authorise the granting of this licence.

For peer review only

Brain MRI and neuropsychological findings at long-term follow-up after COVID-19 hospitalisation: an observational cohort study

Lovisa Hellgren[1](0000-0003-4099-9456), Ulrika Birberg Thornberg[2](0000-0002-6972-3413), Kersti Samuelsson[2](0000-0001-9045-3086), Richard Levi[2](0000-0001-7994-372X), Anestis Divanoglou[2](0000-0001-7376-6793) and Ida Blystad[34](0000-0002-8857-5698)

[1]Department of Rehabilitation Medicine, Region Jönköping County, and Department of Medical and Health Sciences, Linköping University, Linköping, Sweden

[2]Department of Rehabilitation Medicine and Department of Medical and Health Sciences, Linköping University, Linköping, Sweden

[3]Department of Radiology in Linköping and Department of Health, Medicine and Caring Sciences, Linköping University, Linköping, Sweden

[4]Centre for Medical Image Science and Visualization (CMIV), Linköping University, Linköping, Sweden

For peer review only - http://bmjopen.bmj.com/site/about/guidelines.xhtml

OBJECTIVES To report the conjunction of brain MRI and neurocognitive function, as well as persisting fatigue at long-term follow-up after COVID-19 hospitalisation in patients identified as high risk for CNS affection.

DESIGN Ambidirectional observational cohort study.

SETTING All 734 patients from a regional population in Sweden with a laboratory confirmed COVID-19 diagnosis admitted to hospital during the period March 1 to May 31, 2020.

PARTICIPANTS A subgroup (n=185) with persisting symptoms still interfering with daily life at a telephone follow-up at four months post-discharge were invited for a medical and neuropsychological evaluation. Thirty-five out of those who were assessed with a neurocognitive test battery at the clinical visit, and presented a clinical picture concerning for COVID-19-related brain pathology, were further investigated by brain MRI.

MAIN OUTCOME MEASURES Findings on brain MRI, neurocognitive test results and reported fatigue.

RESULTS Twenty-five patients (71%) had abnormalities on MRI; multiple white matter changes were the most common finding. Sixteen patients (46%) demonstrated impaired neurocognitive function, of which ten (29%) with severe impairment. Twenty-six patients (74%) reported clinically significant fatigue. Patients with abnormalities on MRI had a lower visuospatial index (p=0.031) compared with the group with normal MRI.

CONCLUSIONS A majority of patients with concerning post COVID-19 symptoms and/or medical history showed signs of COVID-19 related brain affection detectable by brain MRI and/or neurocognitive test results. Pathological findings were not restricted to patients with severe disease.

Strengths and limitations of this study

- *Clinical characteristics and self-reported long-term outcomes for this cohort were previously presented as part of the LinCoS study.*
- *Participants were examined with MRI about 7 months post admittance to hospital and all MRI images were assessed by an experienced neuroradiologist.*
- *Neurocognitive tests and questionnaires were administered in a standardised way and the evaluation of data was performed by an experienced neuropsychologist.*
- *The lack of a control group for this cohort was a study limitation.*
- *The unavailability of premorbid cognitive testing and MRI needs to be taken into consideration when interpreting the study findings.*

For peer review only - http://bmjopen.bmj.com/site/about/guidelines.xhtml

Introduction

COVID-19 is an infectious disease primarily affecting the respiratory system, but there is accumulating evidence of this virus also affecting the nervous system.[1 2] The exact mechanism behind neurological affection is yet to be elucidated, and whether symptoms depend on direct viral infection or secondary inflammatory effects remains debatable.[1]

Patients with COVID-19 undergoing imaging of the central nervous system (CNS) during the acute phase exhibit various pathological findings.[3] Brain MRI in the acute phase has shown abnormal findings, commonly infarctions, microscopic haemorrhages and/or intra-axial susceptibility abnormalities.[4 5] Lu et al.[6] investigated 60 patients who had recovered from COVID-19 three months after symptom onset. Changes in the microstructure of the white matter as well as in cortical areas pertaining to the olfactory system were reported.[6] To date, very little has been published regarding long-term MRI findings in the brain of patients with COVID-19.

In addition to visualizable pathology by brain MRI, COVID-19 has also been shown to lead to cognitive impairments in several domains, persisting several months following discharge.[7-9] It has been suggested that the severity of the infection (defined in terms of level of medical care needed[10 11] and/or levels of biomarkers of inflammation[7 9]) has a bearing on the degree of resulting neurocognitive impairment as well as findings from MRI.[12]

Our understanding of this disease is still incomplete, particularly regarding the risk for persisting neurocognitive symptoms and potential structural brain damage. The aims of this study were to report the conjunction of brain MRI and neurocognitive function, as well as persisting fatigue at long-term follow-up after COVID-19 hospitalisation in patients identified as high risk for CNS affection.

Methods

This study forms part of the Linköping COVID-19 Study (LinCoS), an ambidirectional observational cohort study.[13] The reporting is informed by the Strengthening the Reporting of Observational studies in Epidemiology (STROBE) statement for cohort studies.[14] LinCoS included all patients (n=734) with a laboratory confirmed COVID-19 diagnosis admitted to hospital for COVID-19 in the total population of Region Östergötland, Sweden, during the period March 1 to May 31, 2020. LinCoS excluded cases with the following characteristics: (1) severe pre-existing comorbidities (such as dementia or under palliative care) making it

For peer review only - http://bmjopen.bmj.com/site/about/guidelines.xhtml

impossible to establish any contribution of COVID-19 to neurocognitive dysfunction; (2) cause for hospitalization unrelated to COVID-19 (such as an operation or labour), and where COVID-19 did not influence medical care, and thus could be deemed coincidental; and (3) those younger than 15 years of age.

All survivors at four months post discharge (n=460) were asked to participate in a structured telephone interview led by one experienced rehabilitation professional (physician, neuropsychologist, occupational therapist, or physiotherapist) focusing on persisting COVID-19 related impairments and activity/participation limitations. Individuals with such problems to such an extent as to significantly impairing daily life (n=185), were then invited to a clinical assessment by a physician and a neuropsychological evaluation by a psychologist. Based on this evaluation, individuals with concerning findings (on neurocognitive testing and/or events during acute hospitalisation indicating possible brain involvement) were referred to brain MRI. Thirty-five individuals who completed a valid neurocognitive assessment and underwent MRI were analysed as part of this study. A flowchart of the inclusion and exclusion is shown in Figure 1. Table 1 presents demographics and background data.

Background data

Premorbid level of function

Premorbid level of function was determined based on patient's performance status and frailty one month preceding COVID-19, as reflected in the medical record in terms of employment, mobility and impact (type, severity and control) of comorbidities. This information was summarised in a modified version of the WHO/ECOG Performance Status[15] and the Frailty Score according to Rockwood[16] to yield an assignment of the premorbid level of function in one of four groups:

1. No or mild frailty: no restriction in daily life activities.

2. Moderate frailty: person mobile and autonomous, but unable to perform physically demanding activities/ work.

3. Considerable frailty: person usually able to perform basic daily life activities, but periodically confined to bed or chair.

4. Severe frailty: person permanently unable to perform activities of daily living and/or confined to bed or chair, eg, dementia necessitating care.

For peer review only - http://bmjopen.bmj.com/site/about/guidelines.xhtml

Individuals in group 4 were excluded from this study according to the LinCoS exclusion criteria described above.

Severity of COVID-19

The WHO Clinical Progression Scale describes patient disease severity by tracking the patient trajectory and resource use over the course of clinical illness.[17] The highest WHO Clinical Progression Scale grade during hospitalisation and/or follow-up time (naming only the categories used in this study) were used:

4. Hospitalised: moderate disease, no oxygen therapy

5. Hospitalised: moderate disease, oxygen by mask or nasal prongs

6. Hospitalised: severe diseases, oxygen by non-invasive ventilation or high flow

7. Hospitalised: severe diseases, intubation and mechanical ventilation, $pO_2/FiO_2 \geq 150$ or $SpO_2/FiO_2 \geq 200$

8. Hospitalised: severe diseases, mechanical ventilation $pO_2/FiO_2 < 150$ ($SpO_2/FiO_2 < 200$) or vasopressors

9. Hospitalised: severe diseases, mechanical ventilation $pO_2/FiO_2 < 150$ and vasopressors, dialysis, or extracorporeal membrane oxygenation

In addition, the maximum values of C-reactive protein (CRP) and D-dimer during hospitalization were retrieved from medical records as additional proxy indicators of systemic disease severity.

Educational level

Information about self-reported formal education were retrieved from the interview performed at 4 months post discharge:

1. Low level of formal education, ≤9 years of school

2. Medium level of formal education, 9 to 12 years of school

3. High level of formal education, >12 years of school

Self-rated health

Data pertaining to self-rated health prior to and following COVID-19 were obtained at the preceding screening interview at four months post-discharge.[13]

For peer review only - http://bmjopen.bmj.com/site/about/guidelines.xhtml

Outcome data

Brain MRI

MRI was performed about seven months (median, 217 days; interquartile range [IQR], 202 to 232 days) post admittance to hospital, and about six and a half months (median, 197 days; IQR, 117 to 258 days) post discharge from hospital. MRI was performed on a clinical GE 3T Architect DV26 MR system according to the following clinical protocol: T2FLAIR ax, T2FSE ax, T1FSE ax, DWI ax, T2FLAIR cor, 3D T1GRE sag, SWI ax. If performed, previous brain MRI or CT examinations were also assessed. MRI images were assessed by a neuroradiologist with ten years of experience.

Neuropsychological evaluation

Patients were assessed by psychologists with long experience from cognitive testing of patients with acquired brain injuries, using neurocognitive tests and questionnaires, approximately five months (median, 142 days; IQR, 120 to 167 days) after discharge from hospital. The time required for a neuropsychological evaluation was approximately one hour (range, 45 to 90 minutes). First, neurocognitive status was assessed using the Repeatable Battery for Assessment of Neuropsychological Status (RBANS).[18] Then the patients completed the Hospital Anxiety and Depression Scale (HADS) and Multidimensional Fatigue Inventory (MFI).[19 20] In four of 35 patients, the neuropsychological evaluation was performed with the assistance of an authorised interpreter. The interpreters were trained in advance for the testing procedure to maintain compliance with the manual guidelines for each test. Only patients with reliable and valid test results were included, as identified through an individual case by case evaluation by members of the research team, resulting in one patient being excluded from interpreter non-compliant. Two patients were excluded due to premorbid diagnoses possibly affecting cognitive function. One patient was excluded due to having less than five years of formal education, thus not complying with the norm group for assessment of test results.[18]

RBANS is a validated battery for assessment of general neurocognitive status. The battery comprises twelve subtests, combined into five different indexes: Immediate Memory index (List Memory and Story Memory subtests), Visuospatial index (Figure Copy and Line Orientation subtests), Language index (Picture Naming and Semantic Fluency subtests), Attention index (Digit Span and Coding subtests), and Delayed Memory index (List Recall,

For peer review only - http://bmjopen.bmj.com/site/about/guidelines.xhtml

List Recognition, Story Recall, and Figure Recall subtests).[18] RBANS total score is achieved by combining the scores of the five indexes. To make comparisons of test results across age spans, raw scores from the neuropsychological tests were transformed into index scores according to the test manual.[18] Each index score ranges from 40 to 160, with lower scores indicating lower performance. The population age-adjusted mean for each index is 100 with a standard deviation (SD) of 15.[18] Test results are presented as the mean and SD, as well as frequencies of results below a cut-off (scores 2SD below the mean according to norms). To determine if the neurocognitive status should be considered impaired, the procedure suggested by Girard et al.[21] and adapted for RBANS by Mitchell et al.[22] was used. Cognition was considered as severely impaired when the patient scored 2SD below the mean according to norms in at least two RBANS indexes, or 1.5SD below the mean according to norms on at least three RBANS indexes. Cognition was considered as mildly/moderately impaired when the patient scored at least 2SD below the mean according to norms on one of the RBANS indexes or 1.5SD below the mean according to norms on two RBANS indexes. When the patient scored higher than stated above, cognition was considered not impaired.

HADS[19] is a valid[19 23] and commonly used questionnaire to detect depression or anxiety. HADS includes 14 questions, categorised into two subscales: depression and anxiety. Each question is scored from 0 to 3, giving a maximum score of 21 for each subscale. In this study, the cut-off for potential cases of depression/anxiety was ≥8, as suggested by Bjelland et al.[23]

The Swedish version of MFI is a valid questionnaire that includes 19 questions focusing on different aspects of fatigue; general fatigue, physical fatigue, mental fatigue, reduced motivation and reduced activity.[20] Each question is scored from 1 to 5 giving a total score ranging from 19 to 95, with higher scores indicating higher levels of fatigue. In this study, the cut-off for clinically significant fatigue was ≥53, as suggested by Hinz et al.[24]

For peer review only - http://bmjopen.bmj.com/site/about/guidelines.xhtml

Statistical analysis

Descriptive results are presented as the mean and SD or median and IQR, as well as frequency. Fisher's exact test was used to compare differences between groups, except for RBANS indexes when Student's *t* test was used. Spearman's correlation coefficient (r_s) was used for correlation analyses. In addition, analysis of variance was used to assess for differences in background data. All statistical tests were two-sided and the accepted level of significance was $P<0.05$. Data were analysed using IBM SPSS Statistics 27.

For peer review only

Results

MRI findings

Twenty-five patients (71%) showed multiple subcortical white matter lesions, located in the cerebral hemispheres near the grey-white matter junction, particularly in the frontal and parietal lobes. Six patients had had MRI performed in the acute phase during their hospitalisation, and all of these patients had additional white matter lesions at the follow-up MRI. Figure 2 shows an example of a patient with new white matter lesions at follow-up, with a typical distribution.

Seven of the patients with pathological MRI had confluent white matter lesions, with a propensity for a biparietal distribution, whereas eight patients had abnormalities on the susceptibility weighted image (SWI), mostly located subcortically, near the grey-white matter junction. Figure 3 shows an example of the SWI pattern in one of the patients. The MRI findings are summarised in supplemental table 1.

Neuropsychological findings

Nineteen of 35 patients (54%) did not show any cognitive impairments according to Mitchell's definition.[22] Six (17%) patients showed mildly/moderately impaired cognition, and ten patients (29%) severely impaired cognition. Immediate and Delayed Memory were the indices where most patients performed below cut-off (2SD below the population based mean) (table 2).

RBANS total score index did not correlate with either age, or time in hospital. Further, there was no significant differences between RBANS total scores regarding WHO Clinical Progression Scale categories, need for ICU care or mechanical ventilator, premorbid level of

function categories, the self-rated health before hospitalisation, the level of education or the need for an interpreter. Cases with missing data in some subtests of RBANS did not differ in background characteristics or outcomes compared to the cases with complete tests (see supplementary table 2).

Clinically significant fatigue (MFI ≥53) was reported by 74% of patients (see table 3). The median scores on the HADS subscales for depression and anxiety were 3.0 (IQR, 1.5 to 5.0) and 4.0 (IQR, 2.0 to 6.5), respectively. Depression (cut-off ≥8) was reported by 14% of patients and anxiety (cut-off ≥8) by 17%.

MRI findings in relation to neuropsychological findings

Patients were divided into two groups depending on whether there were any MRI findings (abnormal MRI) or not (normal MRI). Demographic and background data for the two groups are presented in table 1. Patients with abnormal MRI were older (p=0.007) and had a lower premorbid level of function (p=0.046). There were no between-group differences in days in hospital, need for ICU/mechanical ventilation, CRP or d-dimer levels, or WHO Clinical Progression Scale categories (table 1). The mean value in the Visuospatial Index was lower in the group with abnormal MRI (mean, 81.8; SD, 15.1) compared with the group with normal MRI (mean, 94.3; SD, 11.3) (p=0.031). Otherwise, there were no between-group differences regarding neurocognition, fatigue, depression or anxiety.

Discussion

This study presents brain MRI and neurocognitive findings, as well as self-reported fatigue at long-term follow-up after COVID-19 hospitalisation in patients with clinical risk indicators of higher cerebral dysfunction. The main radiological finding was the presence of multiple white matter changes with a frontal and parietal distribution. This is in line with previous findings on MRI performed during the acute phase of COVID-19.[5] White matter changes are often found with increasing age and can be a marker of cerebrovascular disease. In the present study, the group with abnormal MRI were older, which might be a partial explanation for white matter changes being more frequent in that group. However, all of the patients who had an MRI during the acute phase of COVID-19 had acquired multiple new white matter changes when examined at follow-up. This could indicate that the COVID-19 infection might have an impact on the brain, even in the aftermath of the acute and subacute virus infection.

For peer review only - http://bmjopen.bmj.com/site/about/guidelines.xhtml

For peer review only

Fatigue was reported by 74% of the patients. This is in line with previous reports on fatigue being a common long-term symptom following COVID-19.[25-27] The median score on the MFI subscale General Fatigue at five months follow-up was 15 (IQR, 11 to 18), which should be compared with mean scores at four months follow-up on patients with traumatic brain injury ranging from 10.7 to 12.5 depending on the severity of the traumatic brain injury.[28]

Every fourth patient (29%) had neurocognitive test results corresponding to a severe impairment, whereas half of the patients (54%) did not present any neurocognitive impairment. Because there were no differences regarding cognition (RBANS total scale score) between the patients with different levels of self-rated previous health, premorbid function or level of education, the results might indicate that the impaired cognition detected was caused by the current period of illness rather than any background factors. These findings imply that, in some individuals, a COVID-19 infection might have a negative impact on cognition lasting at least several months post discharge from hospital.

Immediate and Delayed Memory were the indices where most patients scored below cut-off, a finding in agreement with previous research showing impaired results for memory related to COVID-19 infection.[11] Because memory, as well as fatigue, seems to be affected by COVID-19, future research should explore possible interactions with cognition and fatigue following COVID-19.

Few studies have reported associations between MRI findings and neuropsychological functioning following COVID-19.[12] Our results showed that the mean value for the Visuospatial index was significantly lower in the group with abnormal MRI compared with the group with normal MRI. Most patients with abnormal MRI had subcortical white matter lesions, and white matter abnormalities have been shown to be associated with impairment in several cognitive domains.[29 30] The parietal lobes are considered essential for visuospatial functioning, because several neural pathways important for visuospatial processing pass the parietal lobes[31] and visuospatial functions have been attributed to bilateral parietal activation.[32] Thus, the finding in the abnormal MRI group of white matter lesions with a biparietal distribution might be related to the low visuospatial test results seen in this group.

However, there was no significant difference between the two MRI groups regarding the frequency of patients with cognitive impairments or with clinically significant fatigue. This suggests that neither fatigue nor a cognitive impairment after a COVID-19 infection is necessarily captured by presence of abnormal MRI findings in the brain. Thus, when assessing patients with persistent neuropsychological complaints following COVID-19, we

For peer review only - http://bmjopen.bmj.com/site/about/guidelines.xhtml

suggest a multimodal approach including neurocognitive assessment and close evaluation of potential need for MRI.

It has been suggested that there may be an association between days on ventilator care and MRI findings in the brain of patients with COVID-19 in the acute phase.[33] In this cohort, the presence or absence of MRI abnormalities were not associated with ICU care or mechanical ventilation. SWI abnormalities have been reported in the acute phase of COVID-19 infection.[5] In this cohort, SWI abnormalities were found in 8 of 35 cases, which is less frequent than reported from the Stockholm cohort published in 2020[5] where 29 of 39 patients had this finding. However, in the Stockholm cohort study, most of the patients had been on a ventilator in the ICU, whereas in the present cohort, only 54% of the patients needed ventilator care, thus indicating less severe illness in our cohort.

In the present study, there were no significant correlations between RBANS total scale score, time in hospital, or scores on the WHO Clinical Progression Scale. This finding is in contrast to previous studies which suggested that the severity of covid-19 infection has a bearing on the degree of cognitive impairment.[10 11] In the Almeria study, early cognitive testing performed at 10 to 35 days post discharge showed a worse cognitive performance among patients who required oxygen therapy during hospitalisation.[11] In the present study, where neurocognitive assessment was performed five months post discharge, no such association was found.

Results from the present study suggest that MRI findings and/or neurocognitive impairments may occur also in patients with less severe COVID-19, and future research should therefore also include evaluation of patients with COVID-19 who have either been treated in regular pandemic wards or who haven´t been hospitalized at all.

Strengths and limitations

The patients were carefully selected from a population-based cohort study including all confirmed cases of COVID-19 admitted to hospital during a three month period. Brain MRIs and neurocognitive assessments were analysed by an experienced neuroradiologist (IB) and an experienced neuropsychologist (LH) respectively. RBANS is considered a comprehensive test battery[34] and has been recognized as the gold standard neurocognitive battery for diagnosis and clinical trial outcome measurement in Mild Cognitive Impairment.[35] However, RBANS is a relatively brief battery and as such doesn't cover all aspects of cognition. The lack of premorbid MRI makes it difficult to confirm that white matter changes could be solely

For peer review only - http://bmjopen.bmj.com/site/about/guidelines.xhtml

attributed to the COVID-19 infection. It was not manageable to include a control group. It is worth noting, that in cases where an MRI had been performed during the acute phase of the hospitalisation, all patients subsequently had developed new white matter changes, making it plausible to assume that the COVID-19 infection contributed to these white matter changes. This will have an impact on consideration of differential diagnoses when evaluating follow-up MRI on patients who have had COVID-19.

The absence of premorbid cognitive assessment makes it hard to refute that any premorbid factor might have affected the results. However, when considering available background data on premorbid level of functioning and educational level, the poor performance on neurocognitive testing are not to be expected in this cohort. This, in conjunction with frequent patient reports of new impairments in higher cerebral function, point to the likelihood that these results indeed represent COVID-19-related problems.

Conclusions

A majority of patients with concerning neuropsychological symptoms and/or medical history showed signs of COVID-19 related brain affection, detectable by brain MRI and/or neurocognitive test results. Pathological findings were not restricted to patients with severe disease. Thus, post COVID-19 related changes should be considered in the differential diagnosis when white matter changes are detected on brain MRI. Furthermore, it is important to consider post COVID-19 related changes when dealing with patients' reports of neuropsychological deficiency, regardless of severity of disease. As neuropsychological impairments are not always associated with abnormal brain MRI findings, a multi-professional approach would be preferable when assessing patients with persisting neuropsychological complaints following COVID-19.

For peer review only

For peer review only - http://bmjopen.bmj.com/site/about/guidelines.xhtml

References

1. Iadecola C, Anrather J, Kamel H. Effects of COVID-19 on the nervous system. *Cell* 2020
2. Romero-Sánchez CM, Díaz-Maroto I, Fernández-Díaz E, et al. Neurologic manifestations in hospitalized patients with COVID-19: The ALBACOVID registry. *Neurology* 2020;95(8):e1060-e70. doi: 10.1212/wnl.0000000000009937 [published Online First: 2020/06/03]
3. Paterson RW, Brown RL, Benjamin L, et al. The emerging spectrum of COVID-19 neurology: clinical, radiological and laboratory findings. *Brain* 2020 doi: 10.1093/brain/awaa240 [published Online First: 2020/07/09]
4. Chougar L, Shor N, Weiss N, et al. Retrospective Observational Study of Brain MRI Findings in Patients with Acute SARS-CoV-2 Infection and Neurologic Manifestations. *Radiology* 2020;297(3):E313-E23.
5. Klironomos S, Tzortzakakis A, Kits A, et al. Nervous system involvement in COVID-19: results from a retrospective consecutive neuroimaging cohort. *Radiology* 2020:202791.
6. Lu Y, Li X, Geng D, et al. Cerebral micro-structural changes in COVID-19 patients–an MRI-based 3-month follow-up study. *EClinicalMedicine* 2020;25:100484.
7. Mazza MG, Palladini M, De Lorenzo R, et al. Persistent psychopathology and neurocognitive impairment in COVID-19 survivors: effect of inflammatory biomarkers at three-month follow-up. *Brain, behavior, and immunity* 2021;94:138-47.
8. Ferrucci R, Dini M, Groppo E, et al. Long-Lasting Cognitive Abnormalities after COVID-19. *Brain sciences* 2021;11(2) doi: 10.3390/brainsci11020235 [published Online First: 2021/03/07]
9. Miskowiak K, Johnsen S, Sattler S, et al. Cognitive impairments four months after COVID-19 hospital discharge: Pattern, severity and association with illness variables. *European Neuropsychopharmacology* 2021;46:39-48.
10. Negrini F, Ferrario I, Mazziotti D, et al. Neuropsychological Features of Severe Hospitalized Coronavirus Disease 2019 Patients at Clinical Stability and Clues for Postacute Rehabilitation. *Arch Phys Med Rehabil* 2020 doi: 10.1016/j.apmr.2020.09.376 [published Online First: 2020/09/30]
11. Almeria M, Cejudo JC, Sotoca J, et al. Cognitive profile following COVID-19 infection: Clinical predictors leading to neuropsychological impairment. *Brain, behavior, & immunity health* 2020;9:100163. doi: 10.1016/j.bbih.2020.100163 [published Online First: 2020/10/29]
12. Raman B, Cassar MP, Tunnicliffe EM, et al. Medium-term effects of SARS-CoV-2 infection on multiple vital organs, exercise capacity, cognition, quality of life and mental health, post-hospital discharge. *EClinicalMedicine* 2021;31:100683.
13. Divanoglou A, Samuelsson K, Sjödahl R, et al. Rehabilitation needs and mortality associated with the Covid-19 pandemic: a population-based study of all hospitalised and home-healthcare individuals in a Swedish healthcare region. *medRxiv* 2021
14. von Elm E, Altman DG, Egger M, et al. The Strengthening the Reporting of Observational Studies in Epidemiology (STROBE) statement: guidelines for reporting observational studies. *The Lancet* 2007;370(9596):1453-57. doi: 10.1016/S0140-6736(07)61602-X
15. Clegg A, Young J, Iliffe S, et al. Frailty in elderly people. *The Lancet* 2013;381(9868):752-62. doi: 10.1016/S0140-6736(12)62167-9
16. Rockwood K, Song X, MacKnight C, et al. A global clinical measure of fitness and frailty in elderly people. *Cmaj* 2005;173(5):489-95.
17. Marshall JC, Murthy S, Diaz J, et al. A minimal common outcome measure set for COVID-19 clinical research. *The Lancet Infectious Diseases* 2020
18. Randolph C. RBANS update: Repeatable battery for the assessment of neuropsychological status: PsychCorp 2012.
19. Zigmond AS, Snaith RP. The hospital anxiety and depression scale. *Acta psychiatrica scandinavica* 1983;67(6):361-70.

For peer review only

20. Lundh Hagelin C, Wengström Y, Runesdotter S, et al. The psychometric properties of the Swedish Multidimensional Fatigue Inventory MFI-20 in four different populations. *Acta Oncologica* 2007;46(1):97-104.
21. Girard TD, Jackson JC, Pandharipande PP, et al. Delirium as a predictor of long-term cognitive impairment in survivors of critical illness. *Critical care medicine* 2010;38(7):1513.
22. Mitchell ML, Shum DH, Mihala G, et al. Long-term cognitive impairment and delirium in intensive care: A prospective cohort study. *Australian Critical Care* 2018;31(4):204-11.
23. Bjelland I, Dahl AA, Haug TT, et al. The validity of the Hospital Anxiety and Depression Scale: an updated literature review. *Journal of psychosomatic research* 2002;52(2):69-77.
24. Hinz A, Fleischer M, Brähler E, et al. Fatigue in patients with sarcoidosis, compared with the general population. *Gen Hosp Psychiatry* 2011;33(5):462-8. doi: 10.1016/j.genhosppsych.2011.05.009 [published Online First: 2011/07/14]
25. Mandal S, Barnett J, Brill SE, et al. 'Long-COVID': a cross-sectional study of persisting symptoms, biomarker and imaging abnormalities following hospitalisation for COVID-19. *Thorax* 2020 doi: 10.1136/thoraxjnl-2020-215818 [published Online First: 2020/11/12]
26. Xiong Q, Xu M, Li J, et al. Clinical sequelae of COVID-19 survivors in Wuhan, China: a single-centre longitudinal study. *Clinical Microbiology and Infection* 2020;27(1):89-95.
27. Huang C, Huang L, Wang Y, et al. 6-month consequences of COVID-19 in patients discharged from hospital: a cohort study. *The Lancet* 2021
28. Beaulieu-Bonneau S, Ouellet M-C. Fatigue in the first year after traumatic brain injury: course, relationship with injury severity, and correlates. *Neuropsychological rehabilitation* 2017;27(7):983-1001.
29. Gunning-Dixon FM, Raz N. The cognitive correlates of white matter abnormalities in normal aging: a quantitative review. *Neuropsychology* 2000;14(2):224.
30. Filley CM, Fields RD. White matter and cognition: making the connection. *Journal of neurophysiology* 2016;116(5):2093-104.
31. Kravitz DJ, Saleem KS, Baker CI, et al. A new neural framework for visuospatial processing. *Nature Reviews Neuroscience* 2011;12(4):217-30.
32. Seydell-Greenwald A, Ferrara K, Chambers CE, et al. Bilateral parietal activations for complex visual-spatial functions: Evidence from a visual-spatial construction task. *Neuropsychologia* 2017;106:194-206.
33. Agarwal S, Jain R, Dogra S, et al. Cerebral microbleeds and leukoencephalopathy in critically ill patients with COVID-19. *Stroke* 2020;51(9):2649-55.
34. Honarmand K, Lalli RS, Priestap F, et al. Natural history of cognitive impairment in critical illness survivors. A systematic review. *American journal of respiratory and critical care medicine* 2020;202(2):193-201.
35. Karantzoulis S, Novitski J, Gold M, et al. The Repeatable Battery for the Assessment of Neuropsychological Status (RBANS): utility in detection and characterization of mild cognitive impairment due to Alzheimer's disease. *Archives of clinical neuropsychology* 2013;28(8):837-44.

Contributors: LH, KS, AD, RL, UBT and IB took part in planning the study. LH, UBT and IB participated in data collection. LH, IB and KS analysed the data. LH and IB wrote the original draft of the manuscript. LH, KS, AD, RL, UBT and IB reviewed and edited the manuscript. The corresponding author attests that all listed authors meet the authorship

For peer review only - http://bmjopen.bmj.com/site/about/guidelines.xhtml

criteria and that no others meeting the criteria have been omitted. LH is guarantor for the study.

Funding: The core study from which part of the data was collected was funded by the Swedish state under the agreement between the Swedish government and the county councils (the ALF-agreement).

Competing interests: All authors have completed the ICMJE uniform disclosure form and declare: no support from any organisation for the submitted work; no financial relationships with any organisations that might have an interest in the submitted work in the previous three years, no other relationships or activities that could appear to have influenced the submitted work.

Ethical approval: The study has been approved by the Swedish Ethical Review Authority (Dnr 2020-03029 and 2020-04443).

Data sharing: The Public Access to Information and Secrecy Act in Sweden prohibits us from making individual level data publicly available, thus no additional data will be made available.

Transparency: The lead author affirms that this manuscript is an honest, accurate, and transparent account of the study being reported; that no important aspects of the study have been omitted; and that any discrepancies from the study as planned have been explained.

Patient and public involvement: Formally, due to the early stage of the covid-19 pandemic in Sweden, neither patients nor the public were involved in the design or planning of this study. We intend to seek patient and public involvement in the development of an appropriate method of dissemination. Results from the study will be presented to the public through public service. The patient organisation for covid-19 in Sweden will be contacted and the results from the study will be shared. In addition, the results will be presented at relevant medical conferences.

Provenance and peer review: Not commissioned; externally peer reviewed.

Acknowledgement: We thank Lars Valter at Forum Östergötland, Faculty of Medicine and Health Sciences, Linköping University, Linköping, Sweden for statistical advice; and Ninnie Håkansson at the Radiology Department of Linköping University hospital for invaluable assistance with MRI bookings.

For peer review only - http://bmjopen.bmj.com/site/about/guidelines.xhtml

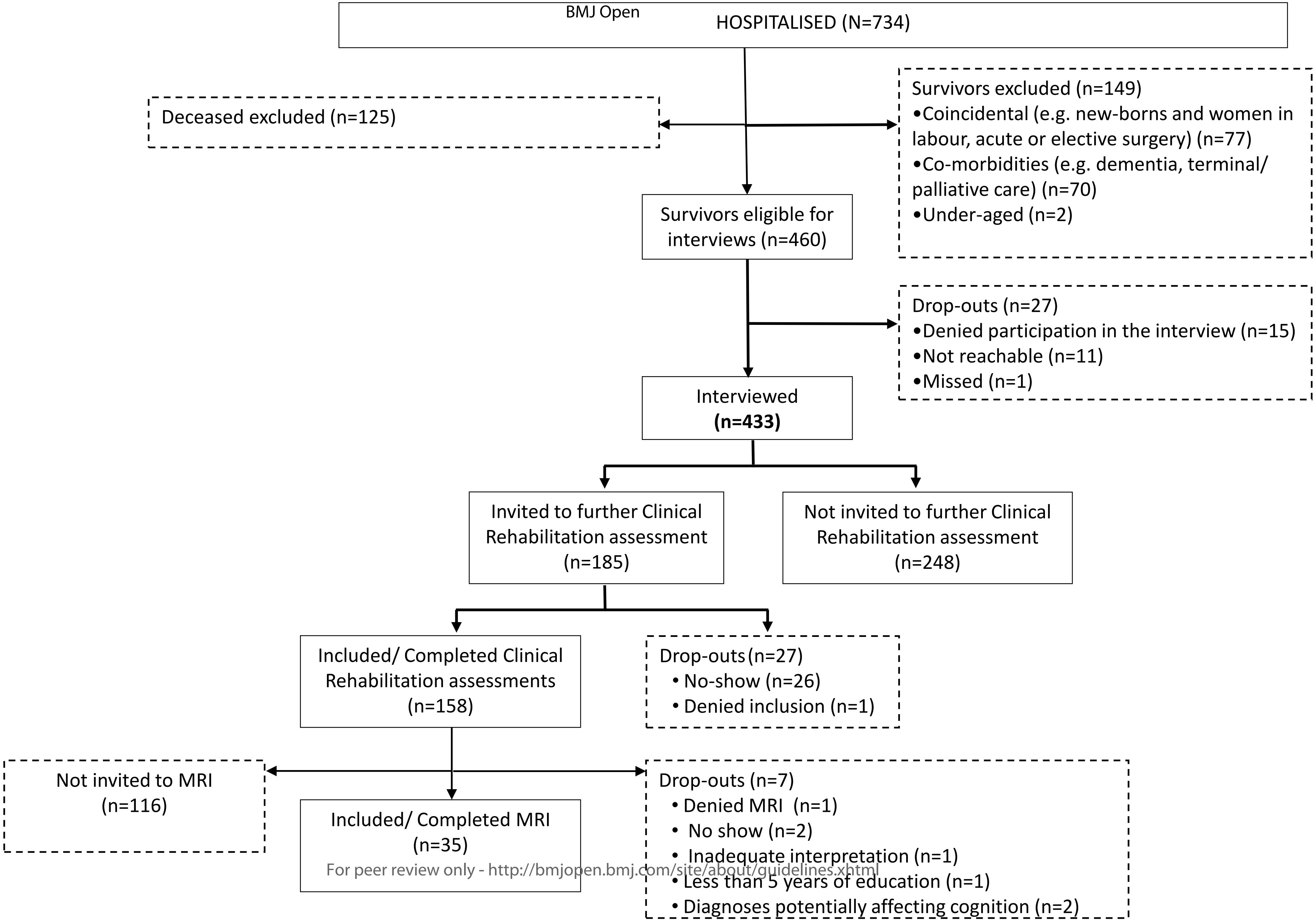

For peer review only - http://bmjopen.bmj.com/site/about/guidelines.xhtml

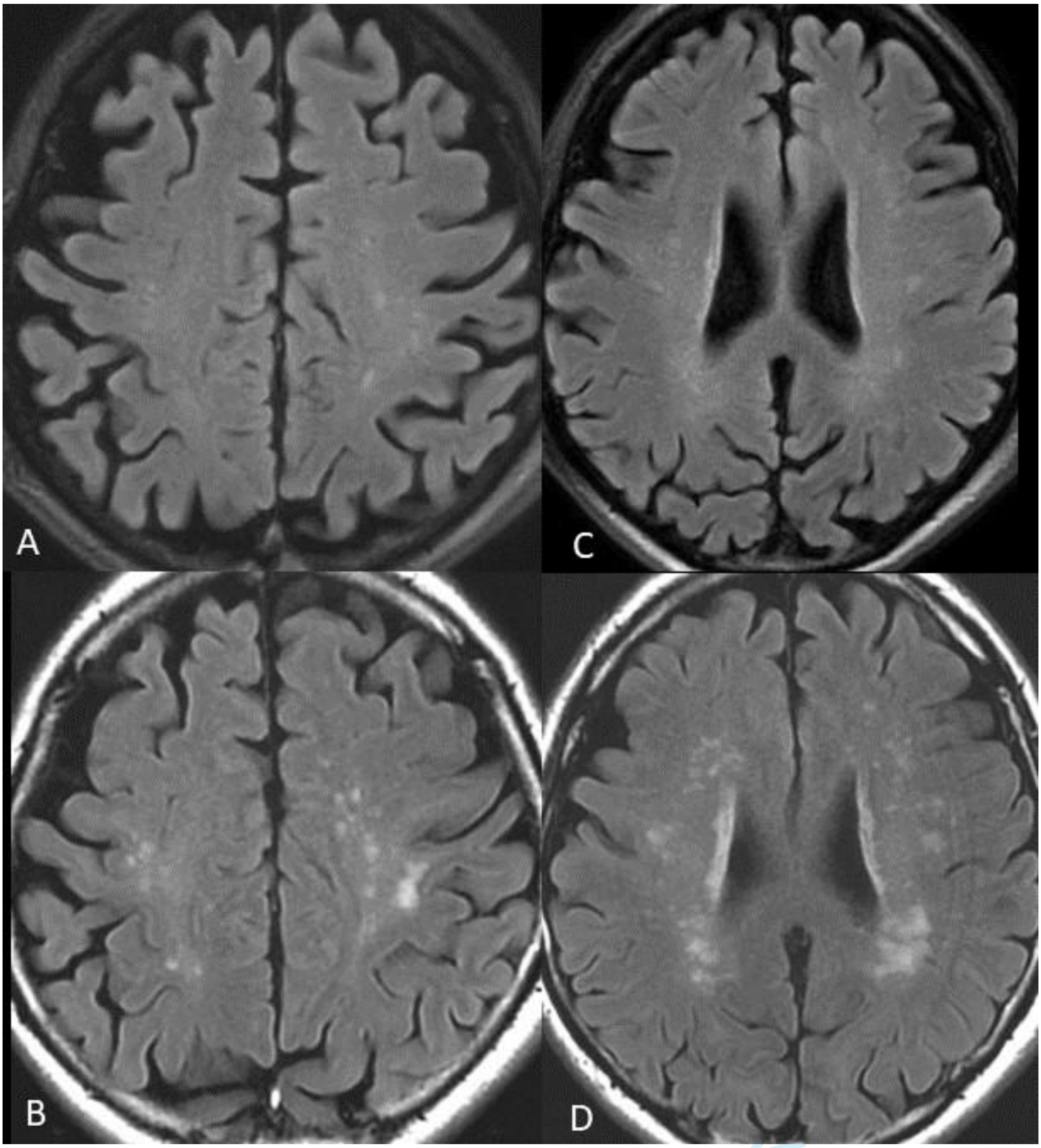

Fig 2 T2FLAIR images for one of the patients. A and C are from the MRI examination performed during the acute phase. B and D are from the MRI examination at late follow-up. New white matter lesions appear at the follow-up MRI, with a typical distribution with a predilection for the parietal and frontal lobes

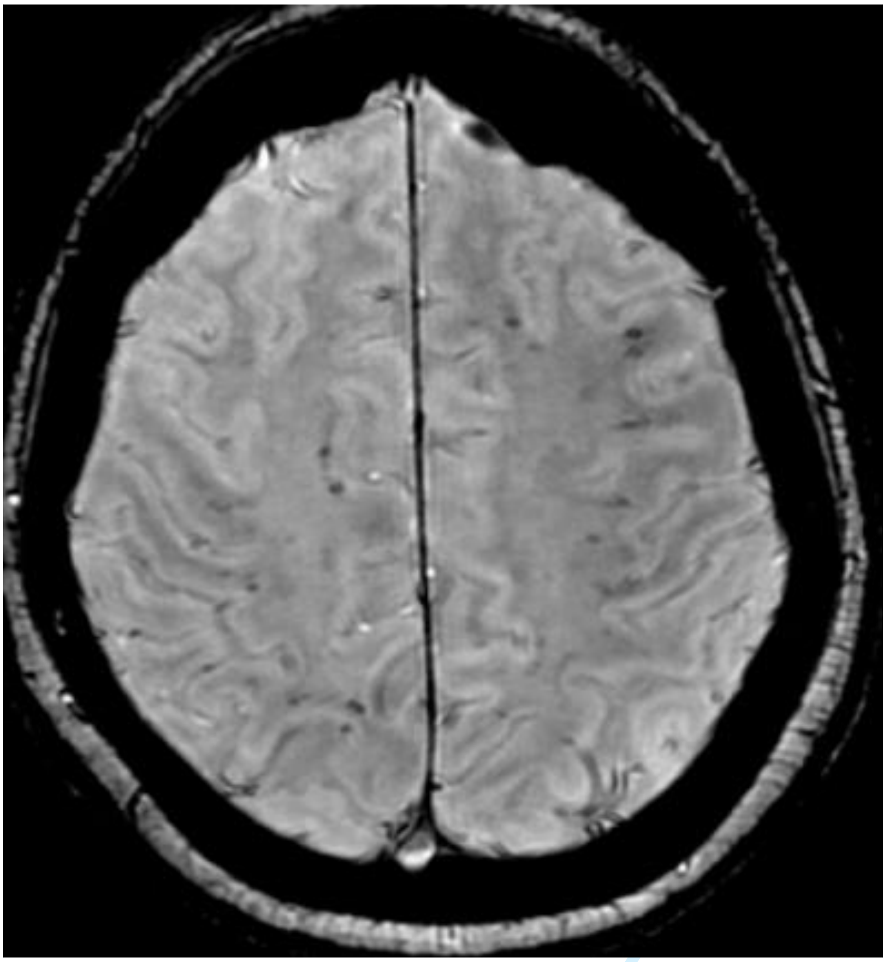

Fig 3 Susceptibility weighted image (SWI) for one of the patients at follow-up MRI. There are multiple SWI abnormalities, mainly located in the grey-white matter junction

For peer review only

Table 1 Characteristics for all included patients previously hospitalised with Covid-19 subdivided into those with abnormal MRI/normal MRI findings

	All included patients (n=35)	Patients with abnormal MRI (n=25)	Patients with normal MRI (n=10)	P value
Age, median (IQR)[a]	59 (51-66)	62.0 (56.0-69.0)	50.5 (47.75-56.5)	0.007
Men/women, n[b]	28/7	21/4	7/3	n.s.
Days in hospital, median (IQR)[a]	18 (7-47)	25 (7-55)	18.0 (8.75-23)	n.s.
ICU care, need/no need, n[b]	20/15	13/12	7/3	n.s.
Mechanical ventilation, need/no need, n[b]	19/14	13/12	6/4	n.s.
Premorbid function category 1/2/3/4, n[b]	20/12/3/0	11/11/3/0	9/1/0/0	0.046
Self-estimated *previous health, n*[b]	13/12/9/0/0	9/9/7/0/0	4/3/2/0/0	n.s.
Self-estimated *present health, n*[b]	4/4/18/8/0	3/4/15/3/0	1/0/3/5/0	n.s.
WHO Clinical Progression Scale 4/5/6/7/8/9, n[b]	4/8/4/0/6/13	2/7/3/0/4/9	2/1/1/0/2/4	n.s.
Educational level 1/2/3, n[b]	10/15/10	9/10/6	1/5/4	n.s.
CRP median (IQR)[a]	202 (144-322)	223 (139-329)	176.5 (119.5-289.5)	n.s.
D-dimer median (IQR)[a]	0.72 (0.38-2.15)	0.76 (0.56-2.28)	0.56 (0.29-1.95)	n.s.

[a]Median test. [b]Fisher test for comparison of frequencies. Categories of premorbid function: 1. No or mild frailty, no restriction in daily life; 2. Moderate frailty, mobile and independent, but unable to handle physically demanding activities or work; 3. Considerable frailty, ability to perform activities of daily living, but in periods confined to bed or chair; and 4. Severe frailty, not able to perform activities of daily living and/or confined to bed or chair. Dementia necessitating care. WHO Clinical Progression Scale: 4. Hospitalised, moderate disease, no oxygen therapy; 5. Hospitalised, moderate disease, oxygen by mask or nasal prongs; 6. Hospitalised, severe diseases, oxygen by non-invasive ventilation or high flow; 7. Hospitalised, severe diseases, intubation and mechanical ventilation, $pO_2/FiO_2 \geq 150$ or $SpO_2/FiO_2 \geq 200$; 8. Hospitalised, severe diseases, mechanical ventilation $pO_2/FiO_2 < 150$ ($SpO_2/FiO_2 < 200$) or vasopressors; and 9. Hospitalised, severe diseases, mechanical ventilation $pO_2/FiO_2 < 150$ and vasopressors, dialysis, or ECMO. Categories of educational level: 1, up to 9 years of school; 2, 9 to 12 years of school; and 3, more than 12 years of school. Categories of self-estimated health (previous and present): 1, Excellent; 2, Very good; 3, Good; 4, Fair; and 5, Poor.

Table 2 RBANS results on patients previously hospitalized with COVID-19

	n	Mean (SD)	Below cut-off (%)
Immediate Memory index	32	89.2 (21.2)	25
Visuospatial index	33	85.2 (15.1)	12
Language index	35	90.9 (16.0)	14
Attention index	34	87.1 (21.4)	9
Delayed Memory index	32	83.9 (18.1)	19
RBANS full-scale index	31	83.0 (17.8)	26

RBANS=The Repeatable Battery for Assessment of Neuropsychological Status. SD=standard deviation. Frequencies of results below cut-off (scores at least 2 standard deviations below the age-adjusted population mean of 100, SD 15 according to the norms) are presented. n=the number of patients who completed subtests needed for the index.

For peer review only

Table 3 Results on MFI on patients previously hospitalized with COVID-19 (n=34)

Subscales of MFI	**Median (IQR)**
General Fatigue (max 20)	15 (11-18)
Physical Fatigue (max 20)	14 (11,5-18)
Mental Fatigue (max 15)	9 (6-12)
Reduced Activity (max 20)	13 (11.5-16.25)
Reduced Motivation (max 20)	10,5 (8-13.25)
MFI full score (max 95)	63 (49.75-70)

MFI=Multidimensional Fatigue Inventory. IQR=interquartile range. n=number of patients who completed the questionnaire.

For peer review only

Supplemental table 2 Background data and data from the clinical assessment on patients who did/did not complete all RBANS subtests

	Patients who completed all RBANS subtests	Patients who did not complete all RBANS subtests	P a)	Patients who completed all RBANS subtests (n)	Patients who did not complete all RBANS subtests (n)	P b)	Patients who completed all RBANS subtests (n)	Patients who did not complete all RBANS subtests (n)
Age, median (IQR)[a]	59 (51-66)	62.5 (51.25-76)	n.s.				31	4
Men/women, n[b]				24/7	4/0	n.s.	31	4
Days in hospital, median (IQR)[a]	18 (8-47)	13 (4.75-86.5)	n.s.				31	4
ICU care, need/no need, n[b]				18/13	2/2	n.s.	31	4
Mechanical ventilation, need/no need, n[b]				17/14	2/2	n.s.	31	4
Premorbid function category 1/2/3/4, n[b]				19/10/2/0	1/2/1/0	n.s.	31	4
WHO Clinical Progression Scale 4/5/6/7/8/9, n[b]				3/7/4/0/6/11	1/1/0/0/0/2	n.s.	31	4
Educational level 1/2/3, n[b]				7/15/9	3/0/4	n.s.	31	4
Self-estimated previous health, n[b]				13/9/8/0/0	0/3/1/0/0	n.s.	30	4
Self-estimated present health, n[b]				4/4/16/6/0	0/0/2/2/0	n.s.	30	4
Distribution of impaired cognitive results, category 1/2/3, n[b]				8/6/17	2/0/2	n.s.	31	4
MFI, above cut-off/not above cut-off, n[b]				23/7	3/1	n.s.	30	4
HADS-depression above/not above cut-off, n[b]				3/28	2/2	n.s.	30	3
HADS-anxiety above/not above cut-off, n[b]				4/27	2/2	n.s.	30	3
MRI findings abnormal/normal, n[b]				22/9	3/1	n.s.	31	4

[a)]Median test [b)]Fisher test for comparison of frequencies. The difference was considered significant at $P<0.05$. n.s.=non-significant. n=number of patients. Categories of premorbid function: 1. No or mild frailty, no restriction in daily life; 2. Moderate frailty, mobile and independent, but unable to handle physically demanding activities or work; 3. Considerable frailty, ability to perform activities of daily living, but with periods confined to bed or chair; and 4. Severe frailty, not able to perform activities of daily living and/or confined to bed or chair. Dementia necessitating care. WHO Clinical Progression Scale: 4. Hospitalised, moderate disease, no oxygen therapy; 5. Hospitalised, moderate disease, oxygen by mask or nasal prongs; 6. Hospitalised, severe diseases, oxygen by non-invasive ventilation or high flow; 7. Hospitalised, severe diseases, intubation and mechanical ventilation, $pO_2/FiO_2 \geq 150$ or $SpO_2/FiO_2 \geq 200$; 8. Hospitalised, severe diseases, mechanical ventilation $pO_2/FiO_2 <150$ ($SpO_2/FiO_2 <200$) or vasopressors; and 9. Hospitalised, severe diseases, mechanical ventilation $pO_2/FiO_2 <150$ and vasopressors, dialysis, or extracorporeal membrane oxygenation. Categories of educational level: 1, up to 9 years of school; 2, 9 to 12 years of school; and 3, more than 12 years of school. Categories of self-estimated health (previous and present): 1, Excellent; 2, Very good; 3, Good; 4, Fair; and 5, Poor. RBANS=The Repeatable Battery for Assessment of Neuropsychological Status Categories of impaired cognitive results: 1, scores 2SD below mean according to norms in at least two RBANS indexes, or 1.5SD below the mean according to the norms on at least three RBANS indexes, was considered severe impairment; 2, scores 2SD below the mean according to the norms on one of the RBANS indexes or 1.5SD below the mean according to the norms on two RBANS indexes, was considered mild/moderate impairment; 3, scores not included in categories 1 and 2, considered not mild/moderate/severe impairment. RBANS cut-off (scores at least 2SDs below age-adjusted population mean of 100, SD 15 according to norms). MFI=Multidimensional Fatigue Inventory. MFI cut-off for clinically significant fatigue ≥53. HADS= Hospital Anxiety and Depression Scale. HADS cut-off for potential depression/anxiety ≥8.

For peer review only

STROBE Statement—Checklist of items that should be included in reports of ***cohort studies***

	Item No	Recommendation	Page No
**Title and abstract**	1	(*a*) Indicate the study's design with a commonly used term in the title or the abstract	2
		(*b*) Provide in the abstract an informative and balanced summary of what was done and what was found	2
**Introduction**			
Background/rationale	2	Explain the scientific background and rationale for the investigation being reported	3
Objectives	3	State specific objectives, including any prespecified hypotheses	3
**Methods**			
Study design	4	Present key elements of study design early in the paper	3-4
Setting	5	Describe the setting, locations, and relevant dates, including periods of recruitment, exposure, follow-up, and data collection	3-4
Participants	6	(*a*) Give the eligibility criteria, and the sources and methods of selection of participants. Describe methods of follow-up	3-4
		(*b*) For matched studies, give matching criteria and number of exposed and unexposed	
Variables	7	Clearly define all outcomes, exposures, predictors, potential confounders, and effect modifiers. Give diagnostic criteria, if applicable	4-7
Data sources/ measurement	8*	For each variable of interest, give sources of data and details of methods of assessment (measurement). Describe comparability of assessment methods if there is more than one group	4-7
Bias	9	Describe any efforts to address potential sources of bias	6, 8
Study size	10	Explain how the study size was arrived at	4
Quantitative variables	11	Explain how quantitative variables were handled in the analyses. If applicable, describe which groupings were chosen and why	7-8
Statistical methods	12	(*a*) Describe all statistical methods, including those used to control for confounding	7-8
		(*b*) Describe any methods used to examine subgroups and interactions	7-8
		(*c*) Explain how missing data were addressed	8
		(*d*) If applicable, explain how loss to follow-up was addressed	n.a.
		(*e*) Describe any sensitivity analyses	
**Results**			
Participants	13*	(a) Report numbers of individuals at each stage of study—eg numbers potentially eligible, examined for eligibility, confirmed eligible, included in the study, completing follow-up, and analysed	Fig1
		(b) Give reasons for non-participation at each stage	Fig1
		(c) Consider use of a flow diagram	Fig1
Descriptive data	14*	(a) Give characteristics of study participants (eg demographic, clinical, social) and information on exposures and potential confounders	Table 1
		(b) Indicate number of participants with missing data for each variable of interest	In each table
		(c) Summarise follow-up time (eg, average and total amount)	6
Outcome data	15*	Report numbers of outcome events or summary measures over time	6-7

For peer review only - http://bmjopen.bmj.com/site/about/guidelines.xhtml

Main results	16	(*a*) Give unadjusted estimates and, if applicable, confounder-adjusted estimates and their precision (eg, 95% confidence interval). Make clear which confounders were adjusted for and why they were included<br>(*b*) Report category boundaries when continuous variables were categorized<br>(*c*) If relevant, consider translating estimates of relative risk into absolute risk for a meaningful time period	8-9
Other analyses	17	Report other analyses done—eg analyses of subgroups and interactions, and sensitivity analyses	8-9
**Discussion**			
Key results	18	Summarise key results with reference to study objectives	9-11
Limitations	19	Discuss limitations of the study, taking into account sources of potential bias or imprecision. Discuss both direction and magnitude of any potential bias	11-12
Interpretation	20	Give a cautious overall interpretation of results considering objectives, limitations, multiplicity of analyses, results from similar studies, and other relevant evidence	9-11
Generalisability	21	Discuss the generalisability (external validity) of the study results	9-11
**Other information**			
Funding	22	Give the source of funding and the role of the funders for the present study and, if applicable, for the original study on which the present article is based	15

*Give information separately for exposed and unexposed groups.

Note: An Explanation and Elaboration article discusses each checklist item and gives methodological background and published examples of transparent reporting. The STROBE checklist is best used in conjunction with this article (freely available on the Web sites of PLoS Medicine at http://www.plosmedicine.org/, Annals of Internal Medicine at http://www.annals.org/, and Epidemiology at http://www.epidem.com/). Information on the STROBE Initiative is available at http://www.strobe-statement.org.

For peer review only

For peer review only - http://bmjopen.bmj.com/site/about/guidelines.xhtml

BMJ Open

Brain MRI and neuropsychological findings at long-term follow-up after COVID-19 hospitalisation: an observational cohort study

Journal:	*BMJ Open*
Manuscript ID	bmjopen-2021-055164.R1
Article Type:	Original research
Date Submitted by the Author:	06-Sep-2021
Complete List of Authors:	Hellgren, Lovisa; Linkoping University Department of Medical and Health Sciences, Department of Rehabilitation Medicine, Region Jönköping County, and Department of Medical and Health Sciences, Linköping University, Linköping, Sweden<br>Birberg Thornberg, Ulrika; Linkoping University Department of Medical and Health Sciences, Department of Rehabilitation Medicine and Department of Medical and Health Sciences, Linköping University, Linköping, Sweden<br>Samuelsson, Kersti; Linkoping University Department of Medical and Health Sciences, Department of Rehabilitation Medicine and Department of Medical and Health Sciences, Linköping University, Linköping, Sweden<br>Levi, Richard; Linkoping University Department of Medical and Health Sciences, Department of Rehabilitation Medicine and Department of Medical and Health Sciences, Linköping University, Linköping, Sweden<br>Divanoglou, Anestis; Linkoping University Department of Medical and Health Sciences, Department of Rehabilitation Medicine and Department of Medical and Health Sciences, Linköping University, Linköping, Sweden<br>Blystad, Ida; Linkoping University Department of Health Medicine and Caring Sciences, Department of Radiology in Linköping and Department of Health, Medicine and Caring Sciences, Linköping University, Linköping, Sweden
**Primary Subject Heading**:	Infectious diseases
Secondary Subject Heading:	Neurology, Rehabilitation medicine
Keywords:	COVID-19, Magnetic resonance imaging < RADIOLOGY & IMAGING, REHABILITATION MEDICINE

SCHOLARONE™
Manuscripts

I, the Submitting Author has the right to grant and does grant on behalf of all authors of the Work (as defined in the below author licence), an exclusive licence and/or a non-exclusive licence for contributions from authors who are: i) UK Crown employees; ii) where BMJ has agreed a CC-BY licence shall apply, and/or iii) in accordance with the terms applicable for US Federal Government officers or employees acting as part of their official duties; on a worldwide, perpetual, irrevocable, royalty-free basis to BMJ Publishing Group Ltd ("BMJ") its licensees and where the relevant Journal is co-owned by BMJ to the co-owners of the Journal, to publish the Work in this journal and any other BMJ products and to exploit all rights, as set out in our licence.

The Submitting Author accepts and understands that any supply made under these terms is made by BMJ to the Submitting Author unless you are acting as an employee on behalf of your employer or a postgraduate student of an affiliated institution which is paying any applicable article publishing charge ("APC") for Open Access articles. Where the Submitting Author wishes to make the Work available on an Open Access basis (and intends to pay the relevant APC), the terms of reuse of such Open Access shall be governed by a Creative Commons licence – details of these licences and which Creative Commons licence will apply to this Work are set out in our licence referred to above.

Other than as permitted in any relevant BMJ Author's Self Archiving Policies, I confirm this Work has not been accepted for publication elsewhere, is not being considered for publication elsewhere and does not duplicate material already published. I confirm all authors consent to publication of this Work and authorise the granting of this licence.

For peer review only

Brain MRI and neuropsychological findings at long-term follow-up after COVID-19 hospitalisation: an observational cohort study

Lovisa Hellgren[1](0000-0003-4099-9456), Ulrika Birberg Thornberg[2](0000-0002-6972-3413), Kersti Samuelsson[2](0000-0001-9045-3086), Richard Levi[2](0000-0001-7994-372X), Anestis Divanoglou[2](0000-0001-7376-6793) and Ida Blystad[34](0000-0002-8857-5698)

[1]Department of Rehabilitation Medicine, Region Jönköping County, and Department of Medical and Health Sciences, Linköping University, Linköping, Sweden

[2]Department of Rehabilitation Medicine and Department of Medical and Health Sciences, Linköping University, Linköping, Sweden

[3]Department of Radiology in Linköping and Department of Health, Medicine and Caring Sciences, Linköping University, Linköping, Sweden

[4]Centre for Medical Image Science and Visualization (CMIV), Linköping University, Linköping, Sweden

Correspondence to: Lovisa Hellgren lovisa.hellgren@liu.se

For peer review only - http://bmjopen.bmj.com/site/about/guidelines.xhtml

OBJECTIVES To report findings on brain MRI and neurocognitive function, as well as persisting fatigue at long-term follow-up after COVID-19 hospitalisation in patients identified as high risk for affection of the central nervous system.

DESIGN Ambidirectional observational cohort study.

SETTING All 734 patients from a regional population in Sweden with a laboratory-confirmed COVID-19 diagnosis admitted to hospital during the period March 1 to May 31, 2020.

PARTICIPANTS A subgroup (n=185) with persisting symptoms still interfering with daily life at a telephone follow-up 4 months post discharge were invited for a medical and neuropsychological evaluation. Thirty-five of those who were assessed with a neurocognitive test battery at the clinical visit, and presented a clinical picture concerning for COVID-19-related brain pathology, were further investigated by brain MRI.

MAIN OUTCOME MEASURES Findings on brain MRI, neurocognitive test results and reported fatigue.

RESULTS Twenty-five patients (71%) had abnormalities on MRI; multiple white matter lesions were the most common finding. Sixteen patients (46%) demonstrated impaired neurocognitive function, of which ten (29%) had severe impairment. Twenty-six patients (74%) reported clinically significant fatigue. Patients with abnormalities on MRI had a lower visuospatial index (p=0.031) compared with the group with normal MRI findings.

CONCLUSIONS In this group of patients selected to undergo MRI after a clinical evaluation, a majority of patients had abnormal MRI and/or neurocognitive test results. Abnormal findings were not restricted to patients with severe disease.

Strengths and limitations of this study

- Detailed clinical characteristics and self-reported long-term outcomes for this cohort were previously presented as part of the LinCoS study.
- Participants were examined with MRI about 7 months after admittance to hospital and all MRI images were assessed by an experienced neuroradiologist.
- Neurocognitive tests and questionnaires were administered in a standardised way and evaluation of the data was performed by experienced neuropsychologists.
- The lack of a control group for this cohort was a study limitation.
- The unavailability of premorbid cognitive testing and MRI needs to be taken into consideration when interpreting the findings.

For peer review only - http://bmjopen.bmj.com/site/about/guidelines.xhtml

Introduction

COVID-19 is an infectious disease primarily affecting the respiratory system, but there is accumulating evidence that this virus also affects the nervous system.[1 2] The exact mechanism behind neurological affection is yet to be elucidated, and whether symptoms depend on direct viral infection or secondary inflammatory effects remains debatable.[1]

Patients with COVID-19 undergoing imaging of the central nervous system (CNS) during the acute phase exhibit various pathological findings.[3] Brain MRI in the acute phase has shown abnormal findings, commonly infarctions, microscopic haemorrhages and/or intra-axial susceptibility abnormalities.[4 5] Lu et al.[6] investigated 60 patients who had recovered from COVID-19 three months after symptom onset. Changes in the microstructure of the white matter as well as in cortical areas pertaining to the olfactory system were reported.[6] To date, very little has been published regarding long-term MRI findings in the brain of patients with COVID-19.

In addition to visualisable pathology by brain MRI, COVID-19 has also been shown to lead to cognitive impairments in several domains, persisting several months after discharge.[7-9] It has been suggested that the severity of the infection (defined in terms of level of medical care needed[10 11] and/or levels of biomarkers of inflammation[7 9]) has a bearing on the degree of resulting neurocognitive impairment as well as findings from MRI.[12]

Our understanding of this disease is still incomplete, particularly regarding the risk for persisting neurocognitive symptoms and potential structural brain damage. The aims of this study were to report the association of brain MRI findings and neurocognitive function, as well as persisting fatigue at long-term follow-up after COVID-19 hospitalisation in patients identified as high risk for CNS affection.

Methods

This study forms part of the Linköping COVID-19 Study (LinCoS), an ambidirectional observational cohort study.[13] The reporting is informed by the Strengthening the Reporting of Observational studies in Epidemiology (STROBE) statement for cohort studies.[14] LinCoS included all patients (n=734) with a laboratory confirmed COVID-19 diagnosis admitted to hospital for COVID-19 in the total population of Region Östergötland, Sweden, during the

For peer review only - http://bmjopen.bmj.com/site/about/guidelines.xhtml

period March 1 to May 31, 2020. LinCoS excluded cases with the following characteristics: (1) severe pre-existing comorbidities (such as dementia or under palliative care) making it impossible to establish any contribution of COVID-19 to neurocognitive dysfunction; (2) cause for hospitalisation unrelated to COVID-19 (such as an operation or labour), and where COVID-19 did not influence medical care, and thus could be deemed coincidental; and (3) those younger than 15 years of age.

All survivors at four months post discharge (n=460) were asked to participate in a structured telephone interview[13] led by one experienced rehabilitation professional (physician, neuropsychologist, occupational therapist, or physiotherapist). The interview comprised 37 questions focusing on persisting COVID-19-related impairments and activity/participation limitations. The questions had the structure of yes/no, and for each yes, the patient was asked to estimate the impact on everyday life using a scale of 1 to 5. Individuals reporting symptoms such as muscle weakness, headache or cognitive problems significantly affecting their daily life (n=185) were then invited to a clinical evaluation, including a medical examination by a physician and a neuropsychological evaluation by a psychologist. Based on this clinical evaluation, individuals with concerning findings (on neurocognitive testing and/or events during acute hospitalisation indicating possible brain involvement) were referred to brain MRI. Thirty-five individuals who completed a valid neurocognitive assessment and underwent MRI were analysed as part of this study. A flowchart of the inclusion and exclusion process is shown in Figure 1. The background data for patients at different stages of the inclusion process are presented in Supplementary Table 1.

Background data

Premorbid level of function

Premorbid level of function was determined based on patient's performance status and frailty one month preceding COVID-19, as reflected in the medical record in terms of employment, mobility and impact (type, severity and control) of comorbidities. This information was summarised in a modified version of the WHO/ECOG Performance Status[15] and the Frailty Score according to Rockwood[16] to yield an assignment of the premorbid level of function in one of four groups:

1. No or mild frailty: no restriction in daily life activities.

For peer review only - http://bmjopen.bmj.com/site/about/guidelines.xhtml

2. Moderate frailty: person mobile and autonomous, but unable to perform physically demanding activities/ work.

3. Considerable frailty: person usually able to perform basic daily life activities, but periodically confined to bed or chair.

4. Severe frailty: person permanently unable to perform activities of daily living and/or confined to bed or chair, eg, dementia necessitating care.

Individuals in group 4 were excluded from this study according to the LinCoS exclusion criteria described above.

Severity of COVID-19

The WHO Clinical Progression Scale describes patient disease severity by tracking the patient trajectory and resource use over the course of clinical illness.[17] The highest WHO Clinical Progression Scale grade during hospitalisation and/or follow-up time (naming only the categories used in this study) were used:

4. Hospitalised: moderate disease, no oxygen therapy

5. Hospitalised: moderate disease, oxygen by mask or nasal prongs

6. Hospitalised: severe diseases, oxygen by non-invasive ventilation or high flow

7. Hospitalised: severe diseases, intubation and mechanical ventilation, $pO_2/FiO_2 \geq 150$ or $SpO_2/FiO_2 \geq 200$

8. Hospitalised: severe diseases, mechanical ventilation $pO_2/FiO_2 < 150$ ($SpO_2/FiO_2 < 200$) or vasopressors

9. Hospitalised: severe diseases, mechanical ventilation $pO_2/FiO_2 < 150$ and vasopressors, dialysis, or extracorporeal membrane oxygenation

In addition, the maximum values of C-reactive protein (CRP) and D-dimer during hospitalisation were retrieved from medical records as additional proxy indicators of systemic disease severity.

For peer review only - http://bmjopen.bmj.com/site/about/guidelines.xhtml

Educational level

Information about self-reported formal education were retrieved from the interview performed at 4 months post discharge:

1. Low level of formal education, ≤9 years of school

2. Medium level of formal education, 9 to 12 years of school

3. High level of formal education, >12 years of school

Self-estimated health

Data pertaining to self-estimated health before and after COVID-19 were obtained at the preceding screening interview at four months post discharge.[13]

Outcome data

Brain MRI

MRI was performed about seven 7 months (median, 217 days; interquartile range [IQR], 202 to 232 days) post admittance to hospital, and about six and a half months (median, 197 days; IQR, 117 to 258 days) post discharge from hospital. MRI was performed on a clinical GE 3T Architect DV26 MR system according to the following clinical protocol: T2FLAIR ax, T2FSE ax, T1FSE ax, DWI ax, T2FLAIR cor, 3D T1GRE sag, SWI ax. If performed, previous brain MRI or CT examinations were also assessed. MRI images were assessed by a neuroradiologist with ten years of experience.

Neuropsychological evaluation

Patients were assessed by psychologists with long experience from cognitive testing of patients with acquired brain injuries, using neurocognitive tests and questionnaires, approximately five months (median, 142 days; IQR, 120 to 167 days) after discharge from hospital. The time required for a neuropsychological evaluation was approximately one hour (range, 45 to 90 minutes). First, neurocognitive status was assessed using the Repeatable Battery for Assessment of Neuropsychological Status (RBANS).[18] Then the patients completed the Hospital Anxiety and Depression Scale (HADS) and Multidimensional Fatigue Inventory (MFI).[19 20] In four of 35 patients, the neuropsychological evaluation was performed

For peer review only - http://bmjopen.bmj.com/site/about/guidelines.xhtml

with the assistance of an authorised interpreter. The interpreters were trained in advance for the testing procedure to maintain compliance with the manual guidelines for each test. Only patients with reliable and valid test results were included, as identified through an individual case by case evaluation by members of the research team, resulting in one patient being excluded from interpreter non-compliant. Two patients were excluded due to premorbid diagnoses possibly affecting cognitive function. One patient was excluded due to having less than five years of formal education, thus not complying with the norm group for assessment of test results.[18]

RBANS is a validated battery for assessment of general neurocognitive status. The battery comprises 12 subtests, combined into five different indexes: Immediate Memory index (List Memory and Story Memory subtests), Visuospatial index (Figure Copy and Line Orientation subtests), Language index (Picture Naming and Semantic Fluency subtests), Attention index (Digit Span and Coding subtests), and Delayed Memory index (List Recall, List Recognition, Story Recall, and Figure Recall subtests).[18] RBANS total score is achieved by combining the scores of the five indexes. To make comparisons of test results across age spans, raw scores from the neuropsychological tests were transformed into index scores according to the test manual.[18] Each index score ranges from 40 to 160, with lower scores indicating lower performance. The population age-adjusted mean for each index is 100 with a standard deviation (SD) of 15.[18] Test results are presented as the mean and SD, as well as frequencies of results below a cut-off (scores 2SD below the mean according to norms). To determine if the neurocognitive status should be considered impaired, the procedure suggested by Girard et al.[21] and adapted for RBANS by Mitchell et al.[22] was used. Cognition was considered as severely impaired when the patient scored 2SD below the mean according to norms in at least two RBANS indexes, or 1.5SD below the mean according to norms on at least three RBANS indexes. Cognition was considered as mildly/moderately impaired when the patient scored at least 2SD below the mean according to norms on one of the RBANS indexes or 1.5SD below the mean according to norms on two RBANS indexes. When the patient scored higher than stated above, cognition was considered not impaired.

HADS[19] is a valid[19 23] and commonly used questionnaire to detect depression or anxiety. HADS includes 14 questions, categorised into two subscales: depression and anxiety. Each

For peer review only - http://bmjopen.bmj.com/site/about/guidelines.xhtml

question is scored from 0 to 3, giving a maximum score of 21 for each subscale. In this study, the cut-off for potential cases of depression/anxiety was ≥8, as suggested by Bjelland et al.[23]

The Swedish version of MFI is a valid questionnaire that includes 19 questions focusing on different aspects of fatigue; general fatigue, physical fatigue, mental fatigue, reduced motivation and reduced activity.[20] Each question is scored from 1 to 5 giving a total score ranging from 19 to 95, with higher scores indicating higher levels of fatigue. In this study, the cut-off for clinically significant fatigue was ≥53, as suggested by Hinz et al.[24]

Statistical analysis

Descriptive results are presented as the mean and SD or median and IQR, as well as frequency. Fisher's exact test was used to compare differences between groups, except for RBANS indexes when Student's *t* test was used. Spearman's correlation coefficient (r_s) was used for correlation analyses. In addition, analysis of variance was used to assess for differences in background data. All statistical tests were two-sided and the accepted level of significance was P<0.05. Data were analysed using IBM SPSS Statistics 27.

Results

A description and comparison of the patients included in the study, subdivided into patients with abnormal versus normal MRI findings, are presented in Table 1.

Table 1 | Characteristics for all patients included in the study, subdivided into those with abnormal MRI/normal MRI findings and previously hospitalised with COVID-19

	**All patients (n=35)**	**Patients with abnormal MRI (n=25)**	**Patients with normal MRI (n=10)**	**P value**
	59 (51-66)	62.0 (56.0-69.0)	50.5 (47.75-56.5)	0.007
Men/women, n[b]	28/7	21/4	7/3	n.s.
Days in hospital, median (IQR)[a]	18 (7-47)	25 (7-55)	18.0 (8.75-23)	n.s.
ICU care, need/no need, n[b]	20/15	13/12	7/3	n.s.
Mechanical ventilation, need/no need, n[b]	19/14	13/12	6/4	n.s.
Premorbid function category 1/2/3/4, n[b]	20/12/3/0	11/11/3/0	9/1/0/0	0.046
Self-estimated previous health, n[b]	13/12/9/0/0	9/9/7/0/0	4/3/2/0/0	n.s.
Self-estimated present health, n[b]	4/4/18/8/0	3/4/15/3/0	1/0/3/5/0	n.s.
WHO Clinical Progression Scale 4/5/6/7/8/9, n[b]	4/8/4/0/6/13	2/7/3/0/4/9	2/1/1/0/2/4	n.s.

For peer review only - http://bmjopen.bmj.com/site/about/guidelines.xhtml

Educational level 1/2/3, n[b]	10/15/10	9/10/6	1/5/4	n.s.
CRP median (IQR)[a]	202 (144-322)	223 (139-329)	176.5 (119.5-289.5)	n.s.
D-dimer median (IQR)[a]	0.72 (0.38-2.15)	0.76 (0.56-2.28)	0.56 (0.29-1.95)	n.s.

[a]Median test. [b]Fisher test for comparison of frequencies. Categories of premorbid function: 1. No or mild frailty, no restriction in daily life; 2. Moderate frailty, mobile and independent, but unable to handle physically demanding activities or work; 3. Considerable frailty, ability to perform activities of daily living, but in periods confined to bed or chair; and 4. Severe frailty, not able to perform activities of daily living and/or confined to bed or chair. Dementia necessitating care. WHO Clinical Progression Scale: 4. Hospitalised, moderate disease, no oxygen therapy; 5. Hospitalised, moderate disease, oxygen by mask or nasal prongs; 6. Hospitalised, severe diseases, oxygen by non-invasive ventilation or high flow; 7. Hospitalised, severe diseases, intubation and mechanical ventilation, $pO_2/FiO_2 \geq 150$ or $SpO_2/FiO_2 \geq 200$; 8. Hospitalised, severe diseases, mechanical ventilation $pO_2/FiO_2 < 150$ ($SpO_2/FiO_2 < 200$) or vasopressors; and 9. Hospitalised, severe diseases, mechanical ventilation $pO_2/FiO_2 < 150$ and vasopressors, dialysis, or ECMO. Categories of educational level: 1, up to 9 years of school; 2, 9 to 12 years of school; and 3, more than 12 years of school. Categories of self-estimated health (previous and present): 1, Excellent; 2, Very good; 3, Good; 4, Fair; and 5, Poor.

For peer review only

MRI findings

Twenty-five patients (71%) showed multiple subcortical white matter lesions, located in the cerebral hemispheres near the grey-white matter junction, particularly in the frontal and parietal lobes. Six patients had had MRI performed in the acute phase during their hospitalisation, and all of these patients had additional white matter lesions at the follow-up MRI. Figure 2 shows an example of a patient with new white matter lesions at follow-up, with a typical distribution.

Seven of the patients with pathological MRI had confluent white matter lesions, with a propensity for a biparietal distribution, whereas eight patients had abnormalities on the susceptibility weighted image (SWI), mostly located subcortically, near the grey-white matter junction. Figure 3 shows an example of the SWI pattern in one of the patients. The MRI findings are summarised in Supplementary Table 2.

Neuropsychological findings

Sixteen of 35 patients (46%) showed cognitive impairments; six of these (17%) showed mildly/moderately impaired cognition, and ten patients (29%) had severely impaired

For peer review only - http://bmjopen.bmj.com/site/about/guidelines.xhtml

cognition according to Mitchell's definition.[22] Results below cut-off were apparent in all cognitive domains. Immediate and Delayed Memory were the indices where most patients performed below cut-off (2SD below the population-based mean) (Table 2).

Table 2 | RBANS results on patients previously hospitalised with COVID-19

	n^{I}	Mean (SD)	Below cut-off, n^{II} (%)
Immediate Memory index	32	89.2 (21.2)	8 (25%)
Visuospatial index	33	85.2 (15.1)	4 (12%)
Language index	35	90.9 (16.0)	5 (14%)
Attention index	34	87.1 (21.4)	3 (9%)
Delayed Memory index	32	83.9 (18.1)	6 (19%)
RBANS total score	31	83.0 (17.8)	8 (26%)

RBANS= Repeatable Battery for Assessment of Neuropsychological Status. SD=standard deviation. Frequencies of results below cut-off (scores at least 2 standard deviations below the age-adjusted population mean of 100, SD 15 according to the norms) are presented. n^{I}=the number of patients who completed subtests needed for the index. n^{II}=the number of patients with results below cut-off.

RBANS total score index did not correlate with either age, or time in hospital. Further, there was no significant differences between RBANS total scores regarding WHO Clinical Progression Scale categories, need for ICU care or mechanical ventilator, premorbid level of function categories, the self-estimated health before hospitalisation, the level of education or the need for an interpreter. Cases with missing data in some subtests of RBANS did not differ in background characteristics or outcomes compared to the cases with complete tests (Supplementary Table 3).

Clinically significant fatigue (MFI ≥53) was reported by 25 patients (74%) (Table 3). The median scores on the HADS subscales for depression and anxiety were 3.0 (IQR, 1.5 to 5.0) and 4.0 (IQR, 2.0 to 6.5), respectively. Depression (cut-off ≥8) was reported by five patients (14%) and anxiety (cut-off ≥8) by six patients (17%).

Table 3 | Results on MFI on patients previously hospitalised with COVID-19 (n=34)

Subscales of MFI	Median (IQR)
General Fatigue (max 20)	15 (11-18)
Physical Fatigue (max 20)	14 (1.,5-18)
Mental Fatigue (max 15)	9 (6-12)
Reduced Activity (max 20)	13 (11.5-16.25)

For peer review only - http://bmjopen.bmj.com/site/about/guidelines.xhtml

Reduced Motivation (max 20)	10,5 (8-13.25)
MFI full score (max 95)	63 (49.75-70)

MFI=Multidimensional Fatigue Inventory. IQR=interquartile range. n=number of patients who completed the questionnaire.

MRI findings in relation to neuropsychological findings

Patients were divided into two groups depending on whether there were any MRI findings (abnormal MRI) or not (normal MRI). Demographic and background data for the two groups are presented in Table 1. Patients with abnormal MRI were older (p=0.007) and had a lower premorbid level of function (p=0.046). There were no between-group differences in days in hospital, need for ICU/mechanical ventilation, CRP or d-dimer levels, or WHO Clinical Progression Scale categories (Table 1). The mean value in the Visuospatial Index was lower in the group with abnormal MRI (mean, 81.8; SD, 15.1) compared with the group with normal MRI (mean, 94.3; SD, 11.3) (p=0.031). Otherwise, there were no between-group differences regarding neurocognition, fatigue, depression or anxiety.

Discussion

This study presents brain MRI and neurocognitive findings, as well as self-reported fatigue at long-term follow-up after COVID-19 hospitalisation in patients with clinical risk indicators of higher cerebral dysfunction. The main radiological finding was the presence of multiple white matter lesions with a frontal and parietal distribution. This is in line with previous findings on MRI performed during the acute phase of COVID-19, where lesions associated with microvascular structures in the white matter have been reported.[5] White matter lesions are often found with increasing age and can be a marker of cerebrovascular disease. In the present study, the group with abnormal MRI were older, which might be a partial explanation for white matter lesions being more frequent in that group. However, a meta-analysis of neurological complications associated with COVID-19 also found that cerebrovascular disease was the most common neurological injury in individuals aged >50 years, occurring in more than 50% of individuals, and could not be explained by increasing age.[25] This suggests that there seems to be an age-associated vulnerability for cerebrovascular disease as a complication of COVID-19 infection. Furthermore, all the patients who underwent MRI during the acute phase of COVID-19 had acquired multiple new white matter lesions when

For peer review only - http://bmjopen.bmj.com/site/about/guidelines.xhtml

examined at follow-up. This suggests that that the COVID-19 infection might have an impact on the brain, even in the aftermath of the acute and subacute virus infection.

Fatigue was reported by 74% (n=25) of the patients. This is in line with previous reports on fatigue being a common long-term symptom following COVID-19.[26-28] The median score on the MFI subscale General Fatigue at five months follow-up was 15 (IQR, 11 to 18), which should be compared with mean scores at four months follow-up on patients with traumatic brain injury ranging from 10.7 to 12.5 depending on the severity of the traumatic brain injury.[29]

Every fourth patient (29%, n=10) had neurocognitive test results corresponding to a severe impairment, whereas half of the patients (54%, n=19) did not present any neurocognitive impairment. Because there were no differences regarding cognition (RBANS total scale score) between the patients with different levels of self-estimated previous health, premorbid function or level of education, the results might indicate that the impaired cognition detected was caused by the current period of illness rather than any background factors. These findings imply that, in some individuals, a COVID-19 infection might have a negative impact on cognition lasting at least several months post discharge from hospital.

Immediate and Delayed Memory were the indices where most patients scored below cut-off, a finding in agreement with previous research showing impaired results for memory related to COVID-19 infection.[11] Because memory, as well as fatigue, seems to be affected by COVID-19, future research should explore possible interactions with cognition and fatigue following COVID-19.

Few studies have reported associations between MRI findings and neuropsychological functioning following COVID-19.[12] Our results showed that the mean value for the Visuospatial index was significantly lower in the group with abnormal MRI compared with the group with normal MRI. The relevance of this result is difficult to interpret. The sample size is small and only four patients (12%) were below the cut-off demarcating impairment. On the other hand, most patients with abnormal MRI had subcortical white matter lesions, and white matter abnormalities have been shown to be associated with impairment in several cognitive domains.[30 31] The parietal lobes are considered essential for visuospatial functioning, because several neural pathways important for visuospatial processing pass the parietal lobes[32] and visuospatial functions have been attributed to bilateral parietal

For peer review only

activation.[33] Thus, the finding in the abnormal MRI group of white matter lesions with a biparietal distribution might be related to the low visuospatial test results seen in this group.

However, there was no significant difference between the two MRI groups regarding the frequency of patients with cognitive impairments or with clinically significant fatigue. This suggests that neither fatigue nor a cognitive impairment after a COVID-19 infection is necessarily captured by presence of abnormal MRI findings in the brain. Thus, when assessing patients with persistent neuropsychological complaints following COVID-19, we suggest a multimodal approach including neurocognitive assessment and close evaluation of potential need for MRI.

It has been suggested that there may be an association between days on ventilator care and MRI findings in the brain of patients with COVID-19 in the acute phase.[34] In this cohort, the presence or absence of MRI abnormalities were not associated with ICU care or mechanical ventilation. SWI abnormalities have been reported in the acute phase of COVID-19 infection.[5] In this cohort, SWI abnormalities were found in 8 of 35 cases, which is less frequent than reported from the Stockholm cohort published in 2020[5] where 29 of 39 patients had this finding. However, in the Stockholm cohort study, most of the patients had been on a ventilator in the ICU, whereas in the present cohort, only 54% of the patients needed ventilator care, thus indicating less severe illness in our cohort.

In the present study, there were no significant correlations between RBANS total scale score, time in hospital, or scores on the WHO Clinical Progression Scale. This finding is in contrast to previous studies which suggested that the severity of COVID-19 infection has a bearing on the degree of cognitive impairment.[10 11] In the Almeria study, early cognitive testing performed at 10 to 35 days post discharge showed a worse cognitive performance among patients who required oxygen therapy during hospitalisation.[11] In the present study, where neurocognitive assessment was performed five months post discharge, no such association was found.

Results from the present study suggest that MRI findings and/or neurocognitive impairments may occur also in patients with less severe COVID-19, and future research should therefore also include evaluation of patients with COVID-19 who have either been treated in regular pandemic wards or who have not been hospitalised at all.

For peer review only - http://bmjopen.bmj.com/site/about/guidelines.xhtml

Strengths and limitations

The patients were carefully selected from a population-based cohort study including all confirmed cases of COVID-19 admitted to hospital during a three month period. Brain MRIs and neurocognitive assessments were analysed by an experienced neuroradiologist and experienced neuropsychologists respectively. RBANS is considered a comprehensive test battery[35] and has been recognised as the gold standard neurocognitive battery for diagnosis and clinical trial outcome measurement in Mild Cognitive Impairment.[36] However, RBANS is a relatively brief battery and as such does not cover all aspects of cognition. The absence of premorbid cognitive assessment makes it hard to refute that any premorbid factor might have affected the results. However, when considering the background data available on the premorbid level of functioning and educational level, the poor performance on neurocognitive testing is unexpected in this cohort. In conjunction with frequent patient reports of new impairments in higher cerebral function, this point to the likelihood that these results indeed represent COVID-19-related problems.

With the lack of premorbid MRI, as well as a relatively small sample size and because it was not manageable to include a control group, it is difficult to confirm that white matter changes could be solely attributed to the COVID-19 infection. However, in cases where an MRI was performed during the acute phase of the hospitalisation, all patients subsequently developed new white matter lesions, making it plausible to assume that the COVID-19 infection contributed to these white matter lesions. This will have an impact on consideration of differential diagnoses when evaluating follow-up MRI on patients who have had COVID-19, especially in younger patients who display white matter lesions on brain MRI after a COVID-19 infection.

Conclusions

A majority of patients with concerning neuropsychological symptoms and/or medical history selected to undergo MRI after a clinical evaluation showed signs of possible COVID-19-related brain affection, detectable by brain MRI and/or neurocognitive test results. Deviating findings were not restricted to patients with severe disease. Thus, post-COVID-19-related changes could be considered in the differential diagnosis when white matter lesions are detected on brain MRI. Furthermore, it is important to consider post-COVID-19-related changes when dealing with patients' reports of neuropsychological deficiency, regardless of

For peer review only - http://bmjopen.bmj.com/site/about/guidelines.xhtml

the severity of disease. As neuropsychological impairments are not always associated with abnormal brain MRI findings, a multi-professional approach would be preferable when assessing patients with persisting neuropsychological complaints following COVID-19.

References

1. Iadecola C, Anrather J, Kamel H. Effects of COVID-19 on the nervous system. *Cell* 2020

2. Romero-Sánchez CM, Díaz-Maroto I, Fernández-Díaz E, et al. Neurologic manifestations in hospitalized patients with COVID-19: The ALBACOVID registry. *Neurology* 2020;95(8):e1060-e70. doi: 10.1212/wnl.0000000000009937 [published Online First: 2020/06/03]

3. Paterson RW, Brown RL, Benjamin L, et al. The emerging spectrum of COVID-19 neurology: clinical, radiological and laboratory findings. *Brain* 2020 doi: 10.1093/brain/awaa240 [published Online First: 2020/07/09]

4. Chougar L, Shor N, Weiss N, et al. Retrospective Observational Study of Brain MRI Findings in Patients with Acute SARS-CoV-2 Infection and Neurologic Manifestations. *Radiology* 2020;297(3):E313-E23.

5. Klironomos S, Tzortzakakis A, Kits A, et al. Nervous system involvement in COVID-19: results from a retrospective consecutive neuroimaging cohort. *Radiology* 2020:202791.

6. Lu Y, Li X, Geng D, et al. Cerebral micro-structural changes in COVID-19 patients–an MRI-based 3-month follow-up study. *EClinicalMedicine* 2020;25:100484.

7. Mazza MG, Palladini M, De Lorenzo R, et al. Persistent psychopathology and neurocognitive impairment in COVID-19 survivors: effect of inflammatory biomarkers at three-month follow-up. *Brain, behavior, and immunity* 2021;94:138-47.

For peer review only - http://bmjopen.bmj.com/site/about/guidelines.xhtml

For peer review only

8. Ferrucci R, Dini M, Groppo E, et al. Long-Lasting Cognitive Abnormalities after COVID-19. *Brain sciences* 2021;11(2) doi: 10.3390/brainsci11020235 [published Online First: 2021/03/07]

9. Miskowiak K, Johnsen S, Sattler S, et al. Cognitive impairments four months after COVID-19 hospital discharge: Pattern, severity and association with illness variables. *European Neuropsychopharmacology* 2021;46:39-48.

10. Negrini F, Ferrario I, Mazziotti D, et al. Neuropsychological Features of Severe Hospitalized Coronavirus Disease 2019 Patients at Clinical Stability and Clues for Postacute Rehabilitation. *Arch Phys Med Rehabil* 2020 doi: 10.1016/j.apmr.2020.09.376 [published Online First: 2020/09/30]

11. Almeria M, Cejudo JC, Sotoca J, et al. Cognitive profile following COVID-19 infection: Clinical predictors leading to neuropsychological impairment. *Brain, behavior, & immunity health* 2020;9:100163. doi: 10.1016/j.bbih.2020.100163 [published Online First: 2020/10/29]

12. Raman B, Cassar MP, Tunnicliffe EM, et al. Medium-term effects of SARS-CoV-2 infection on multiple vital organs, exercise capacity, cognition, quality of life and mental health, post-hospital discharge. *EClinicalMedicine* 2021;31:100683.

13. Divanoglou A, Samuelsson K, Sjödahl R, et al. Rehabilitation needs and mortality associated with the Covid-19 pandemic: a population-based study of all hospitalised and home-healthcare individuals in a Swedish healthcare region. *medRxiv* 2021

14. von Elm E, Altman DG, Egger M, et al. The Strengthening the Reporting of Observational Studies in Epidemiology (STROBE) statement: guidelines for reporting

For peer review only - http://bmjopen.bmj.com/site/about/guidelines.xhtml

observational studies. *The Lancet* 2007;370(9596):1453-57. doi: 10.1016/S0140-6736(07)61602-X

15. Clegg A, Young J, Iliffe S, et al. Frailty in elderly people. *The Lancet* 2013;381(9868):752-62. doi: 10.1016/S0140-6736(12)62167-9

16. Rockwood K, Song X, MacKnight C, et al. A global clinical measure of fitness and frailty in elderly people. *Cmaj* 2005;173(5):489-95.

17. Marshall JC, Murthy S, Diaz J, et al. A minimal common outcome measure set for COVID-19 clinical research. *The Lancet Infectious Diseases* 2020

18. Randolph C. RBANS update: Repeatable battery for the assessment of neuropsychological status: PsychCorp 2012.

19. Zigmond AS, Snaith RP. The hospital anxiety and depression scale. *Acta psychiatrica scandinavica* 1983;67(6):361-70.

20. Lundh Hagelin C, Wengström Y, Runesdotter S, et al. The psychometric properties of the Swedish Multidimensional Fatigue Inventory MFI-20 in four different populations. *Acta Oncologica* 2007;46(1):97-104.

21. Girard TD, Jackson JC, Pandharipande PP, et al. Delirium as a predictor of long-term cognitive impairment in survivors of critical illness. *Critical care medicine* 2010;38(7):1513.

22. Mitchell ML, Shum DH, Mihala G, et al. Long-term cognitive impairment and delirium in intensive care: A prospective cohort study. *Australian Critical Care* 2018;31(4):204-11.

For peer review only

23. Bjelland I, Dahl AA, Haug TT, et al. The validity of the Hospital Anxiety and Depression Scale: an updated literature review. *Journal of psychosomatic research* 2002;52(2):69-77.

24. Hinz A, Fleischer M, Brähler E, et al. Fatigue in patients with sarcoidosis, compared with the general population. *Gen Hosp Psychiatry* 2011;33(5):462-8. doi: 10.1016/j.genhosppsych.2011.05.009 [published Online First: 2011/07/14]

25. Sullivan BN, Fischer T. Age-Associated Neurological Complications of COVID-19: A Systematic Review and Meta-Analysis. *Frontiers in Aging Neuroscience* 2021:374.

26. Mandal S, Barnett J, Brill SE, et al. 'Long-COVID': a cross-sectional study of persisting symptoms, biomarker and imaging abnormalities following hospitalisation for COVID-19. *Thorax* 2020 doi: 10.1136/thoraxjnl-2020-215818 [published Online First: 2020/11/12]

27. Xiong Q, Xu M, Li J, et al. Clinical sequelae of COVID-19 survivors in Wuhan, China: a single-centre longitudinal study. *Clinical Microbiology and Infection* 2020;27(1):89-95.

28. Huang C, Huang L, Wang Y, et al. 6-month consequences of COVID-19 in patients discharged from hospital: a cohort study. *The Lancet* 2021

29. Beaulieu-Bonneau S, Ouellet M-C. Fatigue in the first year after traumatic brain injury: course, relationship with injury severity, and correlates. *Neuropsychological rehabilitation* 2017;27(7):983-1001.

30. Gunning-Dixon FM, Raz N. The cognitive correlates of white matter abnormalities in normal aging: a quantitative review. *Neuropsychology* 2000;14(2):224.

For peer review only - http://bmjopen.bmj.com/site/about/guidelines.xhtml

31. Filley CM, Fields RD. White matter and cognition: making the connection. *Journal of neurophysiology* 2016;116(5):2093-104.

32. Kravitz DJ, Saleem KS, Baker CI, et al. A new neural framework for visuospatial processing. *Nature Reviews Neuroscience* 2011;12(4):217-30.

33. Seydell-Greenwald A, Ferrara K, Chambers CE, et al. Bilateral parietal activations for complex visual-spatial functions: Evidence from a visual-spatial construction task. *Neuropsychologia* 2017;106:194-206.

34. Agarwal S, Jain R, Dogra S, et al. Cerebral microbleeds and leukoencephalopathy in critically ill patients with COVID-19. *Stroke* 2020;51(9):2649-55.

35. Honarmand K, Lalli RS, Priestap F, et al. Natural history of cognitive impairment in critical illness survivors. A systematic review. *American journal of respiratory and critical care medicine* 2020;202(2):193-201.

36. Karantzoulis S, Novitski J, Gold M, et al. The Repeatable Battery for the Assessment of Neuropsychological Status (RBANS): utility in detection and characterization of mild cognitive impairment due to Alzheimer's disease. *Archives of clinical neuropsychology* 2013;28(8):837-44.

Contributors: LH, KS, AD, RL, UBT and IB took part in planning the study. LH, UBT and IB participated in data collection. LH, IB and KS analysed the data. LH and IB wrote the original draft of the manuscript. LH, KS, AD, RL, UBT and IB reviewed and edited the manuscript. The corresponding author attests that all listed authors meet the authorship criteria and that no others meeting the criteria have been omitted. LH is guarantor for the study.

For peer review only - http://bmjopen.bmj.com/site/about/guidelines.xhtml

Funding: The study was funded by the ALF grant, award/grant number N/A. The funder of the study had no role in study design, data collection, data analysis, data interpretation, or writing of the report.

Competing interests: None declared.

Ethical approval: The study has been approved by the Swedish Ethical Review Authority (Dnr 2020-03029 and 2020-04443).

Data sharing: The Public Access to Information and Secrecy Act in Sweden prohibits us from making individual level data publicly available, thus no additional data will be made available.

Transparency: The lead author affirms that this manuscript is an honest, accurate, and transparent account of the study being reported; that no important aspects of the study have been omitted; and that any discrepancies from the study as planned have been explained.

Patient and public involvement: Formally, due to the early stage of the COVID-19 pandemic in Sweden, neither patients nor the public were involved in the design or planning of this study. We intend to seek patient and public involvement in the development of an appropriate method of dissemination. Results from the study will be presented to the public through public service. The patient organisation for COVID-19 in Sweden will be contacted and the results from the study will be shared. In addition, the results will be presented at relevant medical conferences.

Provenance and peer review: Not commissioned; externally peer reviewed.

Acknowledgements: We thank Lars Valter at Forum Östergötland, Faculty of Medicine and Health Sciences, Linköping University, Linköping, Sweden, for statistical advice; and

For peer review only - http://bmjopen.bmj.com/site/about/guidelines.xhtml

Ninnie Håkansson at the Radiology Department of Linköping University hospital for invaluable assistance with MRI bookings. Last, a special acknowledgement to Agnes Andersson, Julia Eriksson, Jacob Lennartsson, Malin Lindh and Mollie Rönn Holmström for valuable assistance with performing the neurocognitive evaluations.

For peer review only

For peer review only - http://bmjopen.bmj.com/site/about/guidelines.xhtml

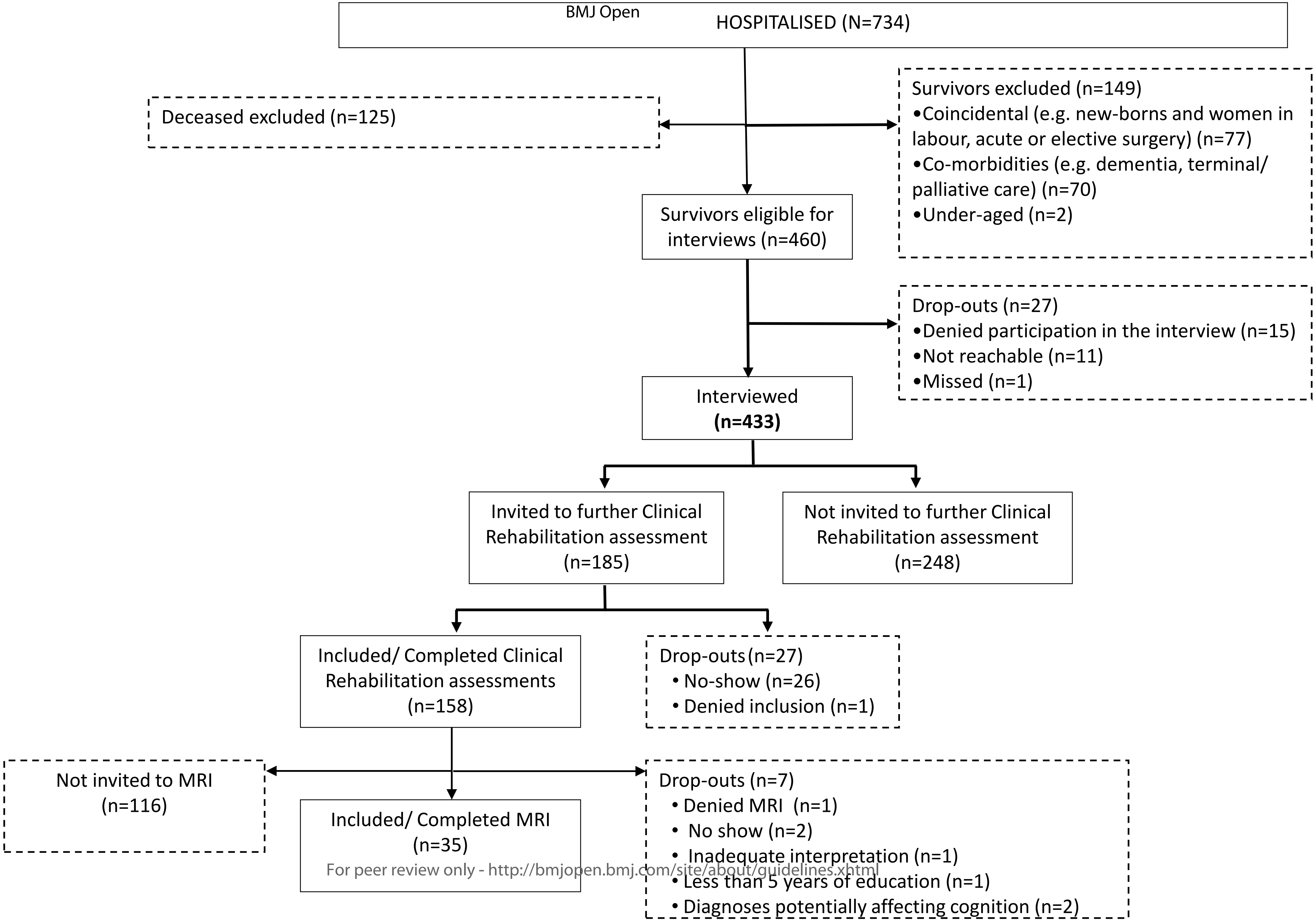

For peer review only - http://bmjopen.bmj.com/site/about/guidelines.xhtml

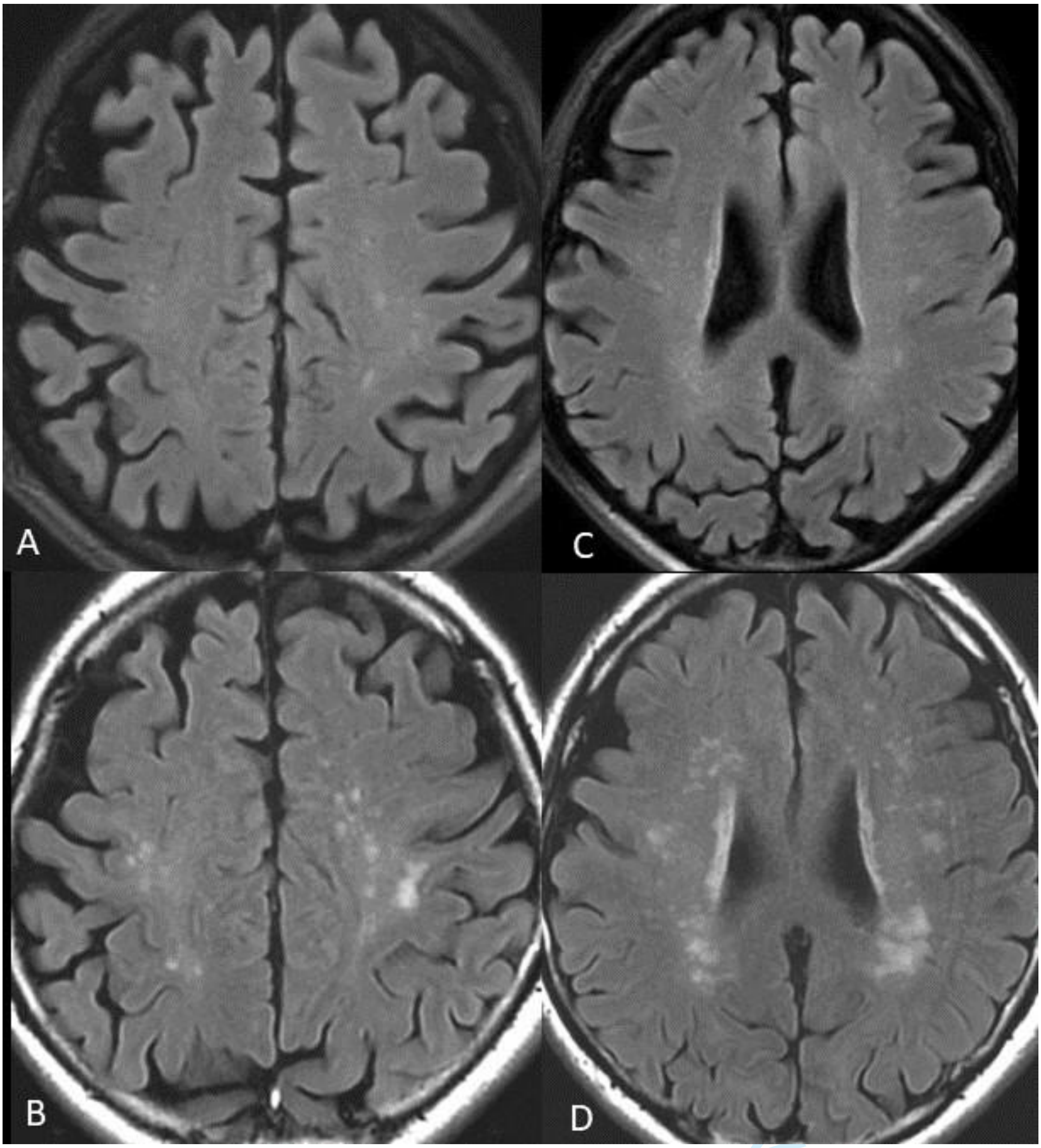

Fig 2 T2FLAIR images for one of the patients. A and C are from the MRI examination performed during the acute phase. B and D are from the MRI examination at late follow-up. New white matter lesions appear at the follow-up MRI, with a typical distribution with a predilection for the parietal and frontal lobes

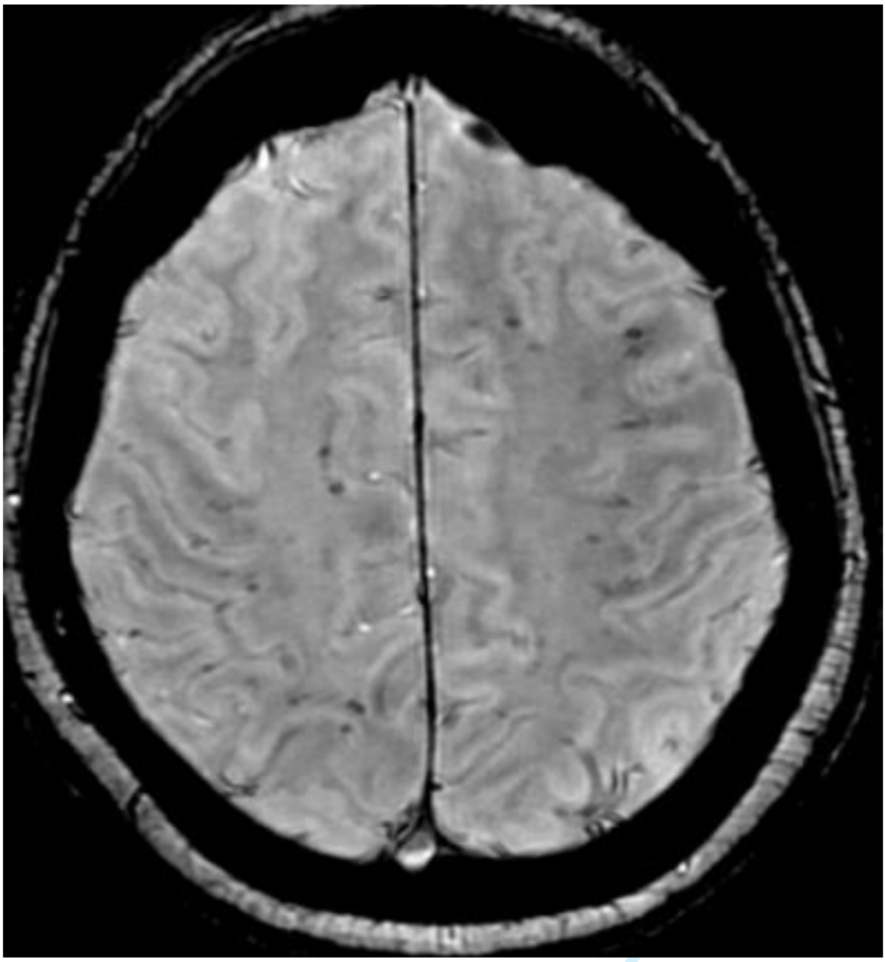

Fig 3 Susceptibility weighted image (SWI) for one of the patients at follow-up MRI. There are multiple SWI abnormalities, mainly located in the grey-white matter junction

For peer review only

Supplemental Table 1. Characteristics for all included patients hospitalised in a Swedish geographical region during the period March 1 to May 31, 2020 with a laboratory confirmed COVID-19; those eligible for interview; those invited for a clinical assessment and those who underwent an MRI

	**All hospitalised (n=734)**	**Eligible for interview (n=460)**	**Invited to a clinical assessment (n=185)**	**MRI performed (n=35)**
Age, median (IQR) years	67 (52-79)	61 (49-74)	59 (49-67)	59 (51-66)
Men/women, n (%)	388 /346 (53/47)	257/203 (56/44)	104/81 (56/44)	28/7 (80/20)
Days in hospital, median (IQR)	6 (3-12)	6 (3-13)	7 (3-19)	18 (7-47)
ICU care, need/no need, n (%)	102/632 (14/86)	72/388 (16/84)	49/136	20/15 (57/43)
Premorbid function category 1/2/3/4/missing, n (%)	210/188/226/100/10 (29/26/31/14/1)	183/142/125/9/1 (40/31/27/2/0)	92/58/32/2/1 (51/31/17/1/1)	20/12/3/0/0 (57/34/9/0/0)
WHO Clinical Progression Scale 4/5/6/7/8/9/10/missing, n (%)	230/261/47/0/25/34/130/7 (31/36/6/0/3/5/18/1)	139/221/41/0/25/34/0/0 (30/48/9/0/5/7/0/0)	51/71/18/0/20/25/0/0 (28/38/10/0/11/14/0/0)	4/8/4/0/6/13/0/0 (11/23/11/0/17/37/0/0)

Categories of premorbid function: 1. No or mild frailty, no restriction in daily life; 2. Moderate frailty, mobile and independent, but unable to handle physically demanding activities or work; 3. Considerable frailty, ability to perform activities of daily living, but in periods confined to bed or chair; and 4. Severe frailty, not able to perform activities of daily living and/or confined to bed or chair. Dementia necessitating care. WHO Clinical Progression Scale: 4. Hospitalised, moderate disease, no oxygen therapy; 5. Hospitalised, moderate disease, oxygen by mask or nasal prongs; 6. Hospitalised, severe diseases, oxygen by non-invasive ventilation or high flow; 7. Hospitalised, severe diseases, intubation and mechanical ventilation, $pO_2/FiO_2 \geq 150$ or $SpO_2/FiO_2 \geq 200$; 8. Hospitalised, severe diseases, mechanical ventilation pO_2/FiO_2 <150 (SpO_2/FiO_2 <200) or vasopressors; and 9. Hospitalised, severe diseases, mechanical ventilation pO_2/FiO_2 <150 and vasopressors, dialysis, or ECMO. Number of symptoms affecting daily life as reported by patients 4 months post discharge.

For peer review only

Supplemental table 2 MRI findings in patients previously hospitalized with COVID-19

Gender	Age (years in intervalls)	Previous MRI in acute phase	SWI abnormalities on MRI	White matter lesions on MRI	Confluent white matter lesions	New white matter lesions	Normal MRI
F	40-49						X
M	40-49						X
M	50-59			X			
F	60-69			X			
M	50-59						X
M	50-59			X	X		
M	40-49						X
M	60-69			X			
M	70-79			X			
F	60-69			X			
M	70-79		X		X		
F	50-59						X
M	60-69	X	X	X	X	X	
M	50-59						X
M	50-59			X			
F	50-59			X			
F	60-69		X				
M	60-69			X	X		
M	50-59			X	X		
M	40-49			X			
M	40-49						X
M	50-59						X
F	60-69						X
M	60-69			X			
M	70-79	X	X	X		X	
M	50-59			X			
M	70-79	X		X	X	X	
M	40-49			X			
M	60-69	X	X	X		X	
M	50-59						X
M	60-69			X			
M	50-59	X		X		X	
M	80-89		X		X		
M	60-69	X	X	X		X	
M	50-59		X				

For peer review only

Supplemental table 3 Background data and data from the clinical assessment on patients who did/did not complete all RBANS subtests

	Patients who completed all RBANS subtests	Patients who did not complete all RBANS subtests	P [a)]	Patients who completed all RBANS subtests (n)	Patients who did not complete all RBANS subtests (n)	P[b)]	Patients who completed all RBANS subtests (n)	Patients who did not complete all RBANS subtests (n)
Age, median (IQR)[a]	59 (51-66)	62.5 (51.25-76)	n.s.				31	4
Men/women, n[b]				24/7	4/0	n.s.	31	4
Days in hospital, median (IQR)[a]	18 (8-47)	13 (4.75-86.5)	n.s.				31	4
ICU care, need/no need, n[b]				18/13	2/2	n.s.	31	4
Mechanical ventilation, need/no need, n[b]				17/14	2/2	n.s.	31	4
Premorbid function category 1/2/3/4, n[b]				19/10/2/0	1/2/1/0	n.s.	31	4
WHO Clinical Progression Scale 4/5/6/7/8/9, n[b]				3/7/4/0/6/11	1/1/0/0/0/2	n.s.	31	4
Educational level 1/2/3, n[b]				7/15/9	3/0/4	n.s.	31	4
Self-estimated previous health, n[b]				13/9/8/0/0	0/3/1/0/0	n.s.	30	4
Self-estimated present health, n[b]				4/4/16/6/0	0/0/2/2/0	n.s.	30	4
Distribution of impaired cognitive results, category 1/2/3, n[b]				8/6/17	2/0/2	n.s.	31	4
MFI, above cut-off/not above cut-off, n[b]				23/7	3/1	n.s.	30	4
HADS-depression above/not above cut-off, n[b]				3/28	2/2	n.s.	30	3
HADS-anxiety above/not above cut-off, n[b]				4/27	2/2	n.s.	30	3
MRI findings abnormal/normal, n[b]				22/9	3/1	n.s.	31	4

a)Median test b)Fisher test for comparison of frequencies. The difference was considered significant at $P<0.05$. n.s.=non-significant. n=number of patients. Categories of premorbid function: 1. No or mild frailty, no restriction in daily life; 2. Moderate frailty, mobile and independent, but unable to handle physically demanding activities or work; 3. Considerable frailty, ability to perform activities of daily living, but with periods confined to bed or chair; and 4. Severe frailty, not able to perform activities of daily living and/or confined to bed or chair. Dementia necessitating care. WHO Clinical Progression Scale: 4. Hospitalised, moderate disease, no oxygen therapy; 5. Hospitalised, moderate disease, oxygen by mask or nasal prongs; 6. Hospitalised, severe diseases, oxygen by non-invasive ventilation or high flow; 7. Hospitalised, severe diseases, intubation and mechanical ventilation, $pO_2/FiO_2 \geq 150$ or $SpO_2/FiO_2 \geq 200$; 8. Hospitalised, severe diseases, mechanical ventilation $pO_2/FiO_2 < 150$ ($SpO_2/FiO_2 < 200$) or vasopressors; and 9. Hospitalised, severe diseases, mechanical ventilation $pO_2/FiO_2 < 150$ and vasopressors, dialysis, or extracorporeal membrane oxygenation. Categories of educational level: 1, up to 9 years of school; 2, 9 to 12 years of school; and 3, more than 12 years of school. Categories of self-estimated health (previous and present): 1, Excellent; 2, Very good; 3, Good; 4, Fair; and 5, Poor. RBANS=The Repeatable Battery for Assessment of Neuropsychological Status Categories of impaired cognitive results: 1, scores 2SD below mean according to norms in at least two RBANS indexes, or 1.5SD below the mean according to the norms on at least three RBANS indexes, was considered severe impairment; 2, scores 2SD below the mean according to the norms on one of the RBANS indexes or 1.5SD below the mean according to the norms on two RBANS indexes, was considered mild/moderate impairment; 3, scores not included in categories 1 and 2, considered not mild/moderate/severe impairment. RBANS cut-off (scores at least 2SDs below age-adjusted population mean of 100, SD 15 according to norms). MFI=Multidimensional Fatigue Inventory. MFI cut-off for clinically significant fatigue ≥53. HADS= Hospital Anxiety and Depression Scale. HADS cut-off for potential depression/anxiety ≥8.

For peer review only

STROBE Statement—Checklist of items that should be included in reports of ***cohort studies***

	Item No	Recommendation	Page No
**Title and abstract**	1	(*a*) Indicate the study's design with a commonly used term in the title or the abstract	2
		(*b*) Provide in the abstract an informative and balanced summary of what was done and what was found	2
**Introduction**			
Background/rationale	2	Explain the scientific background and rationale for the investigation being reported	3
Objectives	3	State specific objectives, including any prespecified hypotheses	3
**Methods**			
Study design	4	Present key elements of study design early in the paper	3-4
Setting	5	Describe the setting, locations, and relevant dates, including periods of recruitment, exposure, follow-up, and data collection	3-4
Participants	6	(*a*) Give the eligibility criteria, and the sources and methods of selection of participants. Describe methods of follow-up	3-4
		(*b*) For matched studies, give matching criteria and number of exposed and unexposed	
Variables	7	Clearly define all outcomes, exposures, predictors, potential confounders, and effect modifiers. Give diagnostic criteria, if applicable	4-7
Data sources/ measurement	8*	For each variable of interest, give sources of data and details of methods of assessment (measurement). Describe comparability of assessment methods if there is more than one group	4-7
Bias	9	Describe any efforts to address potential sources of bias	6, 8
Study size	10	Explain how the study size was arrived at	4
Quantitative variables	11	Explain how quantitative variables were handled in the analyses. If applicable, describe which groupings were chosen and why	7-8
Statistical methods	12	(*a*) Describe all statistical methods, including those used to control for confounding	7-8
		(*b*) Describe any methods used to examine subgroups and interactions	7-8
		(*c*) Explain how missing data were addressed	8
		(*d*) If applicable, explain how loss to follow-up was addressed	n.a.
		(*e*) Describe any sensitivity analyses	
**Results**			
Participants	13*	(a) Report numbers of individuals at each stage of study—eg numbers potentially eligible, examined for eligibility, confirmed eligible, included in the study, completing follow-up, and analysed	Fig1
		(b) Give reasons for non-participation at each stage	Fig1
		(c) Consider use of a flow diagram	Fig1
Descriptive data	14*	(a) Give characteristics of study participants (eg demographic, clinical, social) and information on exposures and potential confounders	Table 1
		(b) Indicate number of participants with missing data for each variable of interest	In each table
		(c) Summarise follow-up time (eg, average and total amount)	6
Outcome data	15*	Report numbers of outcome events or summary measures over time	6-7

For peer review only - http://bmjopen.bmj.com/site/about/guidelines.xhtml

Main results	16	(*a*) Give unadjusted estimates and, if applicable, confounder-adjusted estimates and their precision (eg, 95% confidence interval). Make clear which confounders were adjusted for and why they were included<br>(*b*) Report category boundaries when continuous variables were categorized<br>(*c*) If relevant, consider translating estimates of relative risk into absolute risk for a meaningful time period	8-9
Other analyses	17	Report other analyses done—eg analyses of subgroups and interactions, and sensitivity analyses	8-9
**Discussion**			
Key results	18	Summarise key results with reference to study objectives	9-11
Limitations	19	Discuss limitations of the study, taking into account sources of potential bias or imprecision. Discuss both direction and magnitude of any potential bias	11-12
Interpretation	20	Give a cautious overall interpretation of results considering objectives, limitations, multiplicity of analyses, results from similar studies, and other relevant evidence	9-11
Generalisability	21	Discuss the generalisability (external validity) of the study results	9-11
**Other information**			
Funding	22	Give the source of funding and the role of the funders for the present study and, if applicable, for the original study on which the present article is based	15

*Give information separately for exposed and unexposed groups.

Note: An Explanation and Elaboration article discusses each checklist item and gives methodological background and published examples of transparent reporting. The STROBE checklist is best used in conjunction with this article (freely available on the Web sites of PLoS Medicine at http://www.plosmedicine.org/, Annals of Internal Medicine at http://www.annals.org/, and Epidemiology at http://www.epidem.com/). Information on the STROBE Initiative is available at http://www.strobe-statement.org.

For peer review only

BMJ Open

Brain MRI and neuropsychological findings at long-term follow-up after COVID-19 hospitalisation: an observational cohort study

Journal:	*BMJ Open*
Manuscript ID	bmjopen-2021-055164.R2
Article Type:	Original research
Date Submitted by the Author:	04-Oct-2021
Complete List of Authors:	Hellgren, Lovisa; Linkoping University Department of Medical and Health Sciences, Department of Rehabilitation Medicine, Region Jönköping County, and Department of Medical and Health Sciences, Linköping University, Linköping, Sweden<br>Birberg Thornberg, Ulrika; Linkoping University Department of Medical and Health Sciences, Department of Rehabilitation Medicine and Department of Medical and Health Sciences, Linköping University, Linköping, Sweden<br>Samuelsson, Kersti; Linkoping University Department of Medical and Health Sciences, Department of Rehabilitation Medicine and Department of Medical and Health Sciences, Linköping University, Linköping, Sweden<br>Levi, Richard; Linkoping University Department of Medical and Health Sciences, Department of Rehabilitation Medicine and Department of Medical and Health Sciences, Linköping University, Linköping, Sweden<br>Divanoglou, Anestis; Linkoping University Department of Medical and Health Sciences, Department of Rehabilitation Medicine and Department of Medical and Health Sciences, Linköping University, Linköping, Sweden<br>Blystad, Ida; Linkoping University Department of Health Medicine and Caring Sciences, Department of Radiology in Linköping and Department of Health, Medicine and Caring Sciences, Linköping University, Linköping, Sweden
**Primary Subject Heading**:	Infectious diseases
Secondary Subject Heading:	Neurology, Rehabilitation medicine
Keywords:	COVID-19, Magnetic resonance imaging < RADIOLOGY & IMAGING, REHABILITATION MEDICINE

SCHOLARONE™ Manuscripts

I, the Submitting Author has the right to grant and does grant on behalf of all authors of the Work (as defined in the below author licence), an exclusive licence and/or a non-exclusive licence for contributions from authors who are: i) UK Crown employees; ii) where BMJ has agreed a CC-BY licence shall apply, and/or iii) in accordance with the terms applicable for US Federal Government officers or employees acting as part of their official duties; on a worldwide, perpetual, irrevocable, royalty-free basis to BMJ Publishing Group Ltd ("BMJ") its licensees and where the relevant Journal is co-owned by BMJ to the co-owners of the Journal, to publish the Work in this journal and any other BMJ products and to exploit all rights, as set out in our licence.

The Submitting Author accepts and understands that any supply made under these terms is made by BMJ to the Submitting Author unless you are acting as an employee on behalf of your employer or a postgraduate student of an affiliated institution which is paying any applicable article publishing charge ("APC") for Open Access articles. Where the Submitting Author wishes to make the Work available on an Open Access basis (and intends to pay the relevant APC), the terms of reuse of such Open Access shall be governed by a Creative Commons licence – details of these licences and which Creative Commons licence will apply to this Work are set out in our licence referred to above.

Other than as permitted in any relevant BMJ Author's Self Archiving Policies, I confirm this Work has not been accepted for publication elsewhere, is not being considered for publication elsewhere and does not duplicate material already published. I confirm all authors consent to publication of this Work and authorise the granting of this licence.

For peer review only

Brain MRI and neuropsychological findings at long-term follow-up after COVID-19 hospitalisation: an observational cohort study

Lovisa Hellgren[1](0000-0003-4099-9456), Ulrika Birberg Thornberg[2](0000-0002-6972-3413), Kersti Samuelsson[2](0000-0001-9045-3086), Richard Levi[2](0000-0001-7994-372X), Anestis Divanoglou[2](0000-0001-7376-6793) and Ida Blystad[34](0000-0002-8857-5698)

[1]Department of Rehabilitation Medicine, Region Jönköping County, and Department of Medical and Health Sciences, Linköping University, Linköping, Sweden

[2]Department of Rehabilitation Medicine and Department of Medical and Health Sciences, Linköping University, Linköping, Sweden

[3]Department of Radiology in Linköping and Department of Health, Medicine and Caring Sciences, Linköping University, Linköping, Sweden

[4]Centre for Medical Image Science and Visualization (CMIV), Linköping University, Linköping, Sweden

Correspondence to: Lovisa Hellgren lovisa.hellgren@liu.se

For peer review only - http://bmjopen.bmj.com/site/about/guidelines.xhtml

OBJECTIVES To report findings on brain MRI and neurocognitive function, as well as persisting fatigue at long-term follow-up after COVID-19 hospitalisation in patients identified as high risk for affection of the central nervous system.

DESIGN Ambidirectional observational cohort study.

SETTING All 734 patients from a regional population in Sweden with a laboratory-confirmed COVID-19 diagnosis admitted to hospital during the period March 1 to May 31, 2020.

PARTICIPANTS A subgroup (n=185) with persisting symptoms still interfering with daily life at a telephone follow-up 4 months post discharge were invited for a medical and neuropsychological evaluation. Thirty-five of those who were assessed with a neurocognitive test battery at the clinical visit, and presented a clinical picture concerning for COVID-19-related brain pathology, were further investigated by brain MRI.

MAIN OUTCOME MEASURES Findings on brain MRI, neurocognitive test results and reported fatigue.

RESULTS Twenty-five patients (71%) had abnormalities on MRI; multiple white matter lesions were the most common finding. Sixteen patients (46%) demonstrated impaired neurocognitive function, of which ten (29%) had severe impairment. Twenty-six patients (74%) reported clinically significant fatigue. Patients with abnormalities on MRI had a lower visuospatial index (p=0.031) compared with the group with normal MRI findings.

CONCLUSIONS In this group of patients selected to undergo MRI after a clinical evaluation, a majority of patients had abnormal MRI and/or neurocognitive test results. Abnormal findings were not restricted to patients with severe disease.

For peer review only

Strengths and limitations of this study

- Detailed clinical characteristics and self-reported long-term outcomes for this cohort were previously presented as part of the LinCoS study.
- Participants were examined with MRI about 7 months after admittance to hospital and all MRI images were assessed by an experienced neuroradiologist.
- Neurocognitive tests and questionnaires were administered in a standardised way and evaluation of the data was performed by experienced neuropsychologists.
- The lack of a control group for this cohort was a study limitation.
- The unavailability of premorbid cognitive testing and MRI needs to be taken into consideration when interpreting the findings.

For peer review only - http://bmjopen.bmj.com/site/about/guidelines.xhtml

Introduction

COVID-19 is an infectious disease primarily affecting the respiratory system, but there is accumulating evidence that this virus also affects the nervous system.[1 2] The exact mechanism behind neurological affection is yet to be elucidated, and whether symptoms depend on direct viral infection or secondary inflammatory effects remains debatable.[1]

Patients with COVID-19 undergoing imaging of the central nervous system (CNS) during the acute phase exhibit various pathological findings.[3] Brain MRI in the acute phase has shown abnormal findings, commonly infarctions, microscopic haemorrhages and/or intra-axial susceptibility abnormalities.[4 5] Lu et al.[6] investigated 60 patients who had recovered from COVID-19 three months after symptom onset. Changes in the microstructure of the white matter as well as in cortical areas pertaining to the olfactory system were reported.[6] To date, very little has been published regarding long-term MRI findings in the brain of patients with COVID-19.

In addition to visualisable pathology by brain MRI, COVID-19 has also been shown to lead to cognitive impairments in several domains, persisting several months after discharge.[7-9] It has been suggested that the severity of the infection (defined in terms of level of medical care needed[10 11] and/or levels of biomarkers of inflammation[7 9]) has a bearing on the degree of resulting neurocognitive impairment as well as findings from MRI.[12]

Our understanding of this disease is still incomplete, particularly regarding the risk for persisting neurocognitive symptoms and potential structural brain damage. The aims of this study were to report the association of brain MRI findings and neurocognitive function, as well as persisting fatigue at long-term follow-up after COVID-19 hospitalisation in patients identified as high risk for CNS affection.

Methods

This study forms part of the Linköping COVID-19 Study (LinCoS), an ambidirectional observational cohort study.[13] The reporting is informed by the Strengthening the Reporting of Observational studies in Epidemiology (STROBE) statement for cohort studies.[14] LinCoS included all patients (n=734) with a laboratory confirmed COVID-19 diagnosis admitted to hospital for COVID-19 in the total population of Region Östergötland, Sweden, during the

For peer review only - http://bmjopen.bmj.com/site/about/guidelines.xhtml

period March 1 to May 31, 2020. LinCoS excluded cases with the following characteristics: (1) severe pre-existing comorbidities (such as dementia or under palliative care) making it impossible to establish any contribution of COVID-19 to neurocognitive dysfunction; (2) cause for hospitalisation unrelated to COVID-19 (such as an operation or labour), and where COVID-19 did not influence medical care, and thus could be deemed coincidental; and (3) those younger than 15 years of age.

All survivors at four months post discharge (n=460) were asked to participate in a structured telephone interview[13] led by one experienced rehabilitation professional (physician, neuropsychologist, occupational therapist, or physiotherapist). The interview comprised 37 questions focusing on persisting COVID-19-related impairments and activity/participation limitations. The questions had the structure of yes/no, and for each yes, the patient was asked to estimate the impact on everyday life using a scale of 1 to 5. Individuals reporting symptoms such as muscle weakness, headache or cognitive problems significantly affecting their daily life (n=185) were then invited to a clinical evaluation, including a medical examination by a physician and a neuropsychological evaluation by a psychologist. Based on this clinical evaluation, individuals with concerning findings (on neurocognitive testing and/or events during acute hospitalisation indicating possible brain involvement) were referred to brain MRI. Thirty-five individuals who completed a valid neurocognitive assessment and underwent MRI were analysed as part of this study. A flowchart of the inclusion and exclusion process is shown in Figure 1. The background data for patients at different stages of the inclusion process are presented in Supplementary Table 1.

Background data

Premorbid level of function

Premorbid level of function was determined based on patient's performance status and frailty one month preceding COVID-19, as reflected in the medical record in terms of employment, mobility and impact (type, severity and control) of comorbidities. This information was summarised in a modified version of the WHO/ECOG Performance Status[15] and the Frailty Score according to Rockwood[16] to yield an assignment of the premorbid level of function in one of four groups:

1. No or mild frailty: no restriction in daily life activities.

For peer review only - http://bmjopen.bmj.com/site/about/guidelines.xhtml

2. Moderate frailty: person mobile and autonomous, but unable to perform physically demanding activities/ work.

3. Considerable frailty: person usually able to perform basic daily life activities, but periodically confined to bed or chair.

4. Severe frailty: person permanently unable to perform activities of daily living and/or confined to bed or chair, eg, dementia necessitating care.

Individuals in group 4 were excluded from this study according to the LinCoS exclusion criteria described above.

Severity of COVID-19

The WHO Clinical Progression Scale describes patient disease severity by tracking the patient trajectory and resource use over the course of clinical illness.[17] The highest WHO Clinical Progression Scale grade during hospitalisation and/or follow-up time (naming only the categories used in this study) were used:

4. Hospitalised: moderate disease, no oxygen therapy

5. Hospitalised: moderate disease, oxygen by mask or nasal prongs

6. Hospitalised: severe diseases, oxygen by non-invasive ventilation or high flow

7. Hospitalised: severe diseases, intubation and mechanical ventilation, $pO_2/FiO_2 \geq 150$ or $SpO_2/FiO_2 \geq 200$

8. Hospitalised: severe diseases, mechanical ventilation $pO_2/FiO_2 <150$ ($SpO_2/FiO_2 <200$) or vasopressors

9. Hospitalised: severe diseases, mechanical ventilation $pO_2/FiO_2 <150$ and vasopressors, dialysis, or extracorporeal membrane oxygenation

In addition, the maximum values of C-reactive protein (CRP) and D-dimer during hospitalisation were retrieved from medical records as additional proxy indicators of systemic disease severity.

For peer review only - http://bmjopen.bmj.com/site/about/guidelines.xhtml

Educational level

Information about self-reported formal education were retrieved from the interview performed at 4 months post discharge:

1. Low level of formal education, ≤9 years of school

2. Medium level of formal education, 9 to 12 years of school

3. High level of formal education, >12 years of school

Self-estimated health

Data pertaining to self-estimated health before and after COVID-19 were obtained at the preceding screening interview at four months post discharge.[13]

Outcome data

Brain MRI

MRI was performed about seven 7 months (median, 217 days; interquartile range [IQR], 202 to 232 days) post admittance to hospital, and about six and a half months (median, 197 days; IQR, 117 to 258 days) post discharge from hospital. MRI was performed on a clinical GE 3T Architect DV26 MR system according to the following clinical protocol: T2FLAIR ax, T2FSE ax, T1FSE ax, DWI ax, T2FLAIR cor, 3D T1GRE sag, SWI ax. If performed, previous brain MRI or CT examinations were also assessed. MRI images were assessed by a neuroradiologist with ten years of experience.

Neuropsychological evaluation

Patients were assessed by psychologists with long experience from cognitive testing of patients with acquired brain injuries, using neurocognitive tests and questionnaires, approximately five months (median, 142 days; IQR, 120 to 167 days) after discharge from hospital. The time required for a neuropsychological evaluation was approximately one hour (range, 45 to 90 minutes). First, neurocognitive status was assessed using the Repeatable Battery for Assessment of Neuropsychological Status (RBANS).[18] Then the patients completed the Hospital Anxiety and Depression Scale (HADS) and Multidimensional Fatigue Inventory (MFI).[19,20] In four of 35 patients, the neuropsychological evaluation was performed

For peer review only - http://bmjopen.bmj.com/site/about/guidelines.xhtml

with the assistance of an authorised interpreter. The interpreters were trained in advance for the testing procedure to maintain compliance with the manual guidelines for each test. Only patients with reliable and valid test results were included, as identified through an individual case by case evaluation by members of the research team, resulting in one patient being excluded from interpreter non-compliant. Two patients were excluded due to premorbid diagnoses possibly affecting cognitive function. One patient was excluded due to having less than five years of formal education, thus not complying with the norm group for assessment of test results.[18]

RBANS is a validated battery for assessment of general neurocognitive status. The battery comprises 12 subtests, combined into five different indexes: Immediate Memory index (List Memory and Story Memory subtests), Visuospatial index (Figure Copy and Line Orientation subtests), Language index (Picture Naming and Semantic Fluency subtests), Attention index (Digit Span and Coding subtests), and Delayed Memory index (List Recall, List Recognition, Story Recall, and Figure Recall subtests).[18] RBANS total score is achieved by combining the scores of the five indexes. To make comparisons of test results across age spans, raw scores from the neuropsychological tests were transformed into index scores according to the test manual.[18] Each index score ranges from 40 to 160, with lower scores indicating lower performance. The population age-adjusted mean for each index is 100 with a standard deviation (SD) of 15.[18] Test results are presented as the mean and SD, as well as frequencies of results below a cut-off (scores 2SD below the mean according to norms). To determine if the neurocognitive status should be considered impaired, the procedure suggested by Girard et al.[21] and adapted for RBANS by Mitchell et al.[22] was used. Cognition was considered as severely impaired when the patient scored 2SD below the mean according to norms in at least two RBANS indexes, or 1.5SD below the mean according to norms on at least three RBANS indexes. Cognition was considered as mildly/moderately impaired when the patient scored at least 2SD below the mean according to norms on one of the RBANS indexes or 1.5SD below the mean according to norms on two RBANS indexes. When the patient scored higher than stated above, cognition was considered not impaired.

HADS[19] is a valid[19 23] and commonly used questionnaire to detect depression or anxiety. HADS includes 14 questions, categorised into two subscales: depression and anxiety. Each

For peer review only - http://bmjopen.bmj.com/site/about/guidelines.xhtml

question is scored from 0 to 3, giving a maximum score of 21 for each subscale. In this study, the cut-off for potential cases of depression/anxiety was ≥8, as suggested by Bjelland et al.[23]

The Swedish version of MFI is a valid questionnaire that includes 19 questions focusing on different aspects of fatigue; general fatigue, physical fatigue, mental fatigue, reduced motivation and reduced activity.[20] Each question is scored from 1 to 5 giving a total score ranging from 19 to 95, with higher scores indicating higher levels of fatigue. In this study, the cut-off for clinically significant fatigue was ≥53, as suggested by Hinz et al.[24]

Statistical analysis

Descriptive results are presented as the mean and SD or median and IQR, as well as frequency. Fisher's exact test was used to compare differences between groups, except for RBANS indexes when Student's *t* test was used. Spearman's correlation coefficient (r_s) was used for correlation analyses. In addition, analysis of variance was used to assess for differences in background data. All statistical tests were two-sided and the accepted level of significance was $P<0.05$. The reported p-values were not adjusted for multiple comparisons, due to the increased likelihood of a type II error[25]. Data were analysed using IBM SPSS Statistics 27.

Results

A description and comparison of the patients included in the study, subdivided into patients with abnormal versus normal MRI findings, are presented in Table 1.

Table 1 | Characteristics for all patients included in the study, subdivided into those with abnormal MRI/normal MRI findings and previously hospitalised with COVID-19

	All patients (n=35)	Patients with abnormal MRI (n=25)	Patients with normal MRI (n=10)	P value
Age, median (IQR) years	59 (51-66)	62.0 (56.0-69.0)	50.5 (47.75-56.5)	0.007
Men/women, n[b]	28/7	21/4	7/3	n.s.
Days in hospital, median (IQR)[a]	18 (7-47)	25 (7-55)	18.0 (8.75-23)	n.s.
ICU care, need/no need, n[b]	20/15	13/12	7/3	n.s.
Mechanical ventilation, need/no need, n[b]	19/14	13/12	6/4	n.s.
Premorbid function category 1/2/3/4, n[b]	20/12/3/0	11/11/3/0	9/1/0/0	0.046
Self-estimated previous health, n[b]	13/12/9/0/0	9/9/7/0/0	4/3/2/0/0	n.s.

For peer review only - http://bmjopen.bmj.com/site/about/guidelines.xhtml

Self-estimated present health, n[b]	4/4/18/8/0	3/4/15/3/0	1/0/3/5/0	n.s.
WHO Clinical Progression Scale 4/5/6/7/8/9, n[b]	4/8/4/0/6/13	2/7/3/0/4/9	2/1/1/0/2/4	n.s.
Educational level 1/2/3, n[b]	10/15/10	9/10/6	1/5/4	n.s.
CRP median (IQR)[a]	202 (144-322)	223 (139-329)	176.5 (119.5-289.5)	n.s.
D-dimer median (IQR)[a]	0.72 (0.38-2.15)	0.76 (0.56-2.28)	0.56 (0.29-1.95)	n.s.

[a]Median test. [b]Fisher test for comparison of frequencies. Categories of premorbid function: 1. No or mild frailty, no restriction in daily life; 2. Moderate frailty, mobile and independent, but unable to handle physically demanding activities or work; 3. Considerable frailty, ability to perform activities of daily living, but in periods confined to bed or chair; and 4. Severe frailty, not able to perform activities of daily living and/or confined to bed or chair. Dementia necessitating care. WHO Clinical Progression Scale: 4. Hospitalised, moderate disease, no oxygen therapy; 5. Hospitalised, moderate disease, oxygen by mask or nasal prongs; 6. Hospitalised, severe diseases, oxygen by non-invasive ventilation or high flow; 7. Hospitalised, severe diseases, intubation and mechanical ventilation, $pO_2/FiO_2 \geq 150$ or $SpO_2/FiO_2 \geq 200$; 8. Hospitalised, severe diseases, mechanical ventilation $pO_2/FiO_2 < 150$ ($SpO_2/FiO_2 < 200$) or vasopressors; and 9. Hospitalised, severe diseases, mechanical ventilation $pO_2/FiO_2 < 150$ and vasopressors, dialysis, or ECMO. Categories of educational level: 1, up to 9 years of school; 2, 9 to 12 years of school; and 3, more than 12 years of school. Categories of self-estimated health (previous and present): 1, Excellent; 2, Very good; 3, Good; 4, Fair; and 5, Poor.

MRI findings

Twenty-five patients (71%) showed multiple subcortical white matter lesions, located in the cerebral hemispheres near the grey-white matter junction, particularly in the frontal and parietal lobes. Six patients had had MRI performed in the acute phase during their hospitalisation, and all of these patients had additional white matter lesions at the follow-up MRI. Figure 2 shows an example of a patient with new white matter lesions at follow-up, with a typical distribution.

Seven of the patients with pathological MRI had confluent white matter lesions, with a propensity for a biparietal distribution, whereas eight patients had abnormalities on the susceptibility weighted image (SWI), mostly located subcortically, near the grey-white matter junction. Figure 3 shows an example of the SWI pattern in one of the patients. The MRI findings are summarised in Supplementary Table 2.

For peer review only

Neuropsychological findings

Sixteen of 35 patients (46%) showed cognitive impairments; six of these (17%) showed mildly/moderately impaired cognition, and ten patients (29%) had severely impaired cognition according to Mitchell's definition.[22] Results below cut-off were apparent in all cognitive domains. Immediate and Delayed Memory were the indices where most patients performed below cut-off (2SD below the population-based mean) (Table 2).

Table 2 | RBANS results on patients previously hospitalised with COVID-19

	n^{I}	Mean (SD)	Below cut-off, n^{II} (%)
Immediate Memory index	32	89.2 (21.2)	8 (25%)
Visuospatial index	33	85.2 (15.1)	4 (12%)
Language index	35	90.9 (16.0)	5 (14%)
Attention index	34	87.1 (21.4)	3 (9%)
Delayed Memory index	32	83.9 (18.1)	6 (19%)
RBANS total score	31	83.0 (17.8)	8 (26%)

RBANS= Repeatable Battery for Assessment of Neuropsychological Status. SD=standard deviation. Frequencies of results below cut-off (scores at least 2 standard deviations below the age-adjusted population mean of 100, SD 15 according to the norms) are presented. n^{I}=the number of patients who completed subtests needed for the index. n^{II}=the number of patients with results below cut-off.

RBANS total score index did not correlate with either age, or time in hospital. Further, there was no significant differences between RBANS total scores regarding WHO Clinical Progression Scale categories, need for ICU care or mechanical ventilator, premorbid level of function categories, the self-estimated health before hospitalisation, the level of education or the need for an interpreter. Cases with missing data in some subtests of RBANS did not differ in background characteristics or outcomes compared to the cases with complete tests (Supplementary Table 3).

Clinically significant fatigue (MFI ≥53) was reported by 25 patients (74%) (Table 3). The median scores on the HADS subscales for depression and anxiety were 3.0 (IQR, 1.5 to 5.0) and 4.0 (IQR, 2.0 to 6.5), respectively. Depression (cut-off ≥8) was reported by five patients (14%) and anxiety (cut-off ≥8) by six patients (17%).

Table 3 | Results on MFI on patients previously hospitalised with COVID-19 (n=34)

Subscales of MFI	Median (IQR)
General Fatigue (max 20)	15 (11-18)

For peer review only - http://bmjopen.bmj.com/site/about/guidelines.xhtml

Physical Fatigue (max 20)	14 (1.,5-18)
Mental Fatigue (max 15)	9 (6-12)
Reduced Activity (max 20)	13 (11.5-16.25)
Reduced Motivation (max 20)	10,5 (8-13.25)
MFI full score (max 95)	63 (49.75-70)

MFI=Multidimensional Fatigue Inventory. IQR=interquartile range. n=number of patients who completed the questionnaire.

MRI findings in relation to neuropsychological findings

Patients were divided into two groups depending on whether there were any MRI findings (abnormal MRI) or not (normal MRI). Demographic and background data for the two groups are presented in Table 1. Patients with abnormal MRI were older (p=0.007) and had a lower premorbid level of function (p=0.046). There were no between-group differences in days in hospital, need for ICU/mechanical ventilation, CRP or d-dimer levels, or WHO Clinical Progression Scale categories (Table 1). The mean value in the Visuospatial Index was lower in the group with abnormal MRI (mean, 81.8; SD, 15.1) compared with the group with normal MRI (mean, 94.3; SD, 11.3) (p=0.031). Otherwise, there were no between-group differences regarding neurocognition, fatigue, depression or anxiety.

Discussion

This study presents brain MRI and neurocognitive findings, as well as self-reported fatigue at long-term follow-up after COVID-19 hospitalisation in patients with clinical risk indicators of higher cerebral dysfunction. The main radiological finding was the presence of multiple white matter lesions with a frontal and parietal distribution. This is in line with previous findings on MRI performed during the acute phase of COVID-19, where lesions associated with microvascular structures in the white matter have been reported.[5] White matter lesions are often found with increasing age and can be a marker of cerebrovascular disease. In the present study, the group with abnormal MRI were older, which might be a partial explanation for white matter lesions being more frequent in that group. However, a meta-analysis of neurological complications associated with COVID-19 also found that cerebrovascular disease was the most common neurological injury in individuals aged >50 years, occurring in more than 50% of individuals, and could not be explained by increasing age.[26] This suggests that there seems to be an age-associated vulnerability for cerebrovascular disease as a

For peer review only - http://bmjopen.bmj.com/site/about/guidelines.xhtml

complication of COVID-19 infection. Furthermore, all the patients who underwent MRI during the acute phase of COVID-19 had acquired multiple new white matter lesions when examined at follow-up. This suggests that that the COVID-19 infection might have an impact on the brain, even in the aftermath of the acute and subacute virus infection.

Fatigue was reported by 74% (n=25) of the patients. This is in line with previous reports on fatigue being a common long-term symptom following COVID-19.[27-29] The median score on the MFI subscale General Fatigue at five months follow-up was 15 (IQR, 11 to 18), which should be compared with mean scores at four months follow-up on patients with traumatic brain injury ranging from 10.7 to 12.5 depending on the severity of the traumatic brain injury.[30]

Every fourth patient (29%, n=10) had neurocognitive test results corresponding to a severe impairment, whereas half of the patients (54%, n=19) did not present any neurocognitive impairment. Because there were no differences regarding cognition (RBANS total scale score) between the patients with different levels of self-estimated previous health, premorbid function or level of education, the results might indicate that the impaired cognition detected was caused by the current period of illness rather than any background factors. These findings imply that, in some individuals, a COVID-19 infection might have a negative impact on cognition lasting at least several months post discharge from hospital.

Immediate and Delayed Memory were the indices where most patients scored below cut-off, a finding in agreement with previous research showing impaired results for memory related to COVID-19 infection.[11] Because memory, as well as fatigue, seems to be affected by COVID-19, future research should explore possible interactions with cognition and fatigue following COVID-19.

Few studies have reported associations between MRI findings and neuropsychological functioning following COVID-19.[12] Our results showed that the mean value for the Visuospatial index was significantly lower in the group with abnormal MRI compared with the group with normal MRI. The relevance of this result is difficult to interpret. The p-value was not corrected for multiple comparisons, the sample size was small and only four patients (12%) were below the cut-off demarcating impairment. On the other hand, most patients with abnormal MRI had subcortical white matter lesions, and white matter abnormalities have been shown to be associated with impairment in several cognitive domains.[31 32] The parietal

For peer review only - http://bmjopen.bmj.com/site/about/guidelines.xhtml

lobes are considered essential for visuospatial functioning, because several neural pathways important for visuospatial processing pass the parietal lobes[33] and visuospatial functions have been attributed to bilateral parietal activation.[34] Thus, the finding in the abnormal MRI group of white matter lesions with a biparietal distribution might be related to the low visuospatial test results seen in this group.

However, there was no significant difference between the two MRI groups regarding the frequency of patients with cognitive impairments or with clinically significant fatigue. This suggests that neither fatigue nor a cognitive impairment after a COVID-19 infection is necessarily captured by presence of abnormal MRI findings in the brain. Thus, when assessing patients with persistent neuropsychological complaints following COVID-19, we suggest a multimodal approach including neurocognitive assessment and close evaluation of potential need for MRI.

It has been suggested that there may be an association between days on ventilator care and MRI findings in the brain of patients with COVID-19 in the acute phase.[35] In this cohort, the presence or absence of MRI abnormalities were not associated with ICU care or mechanical ventilation. The inclusion of patients for MRI in this study might have affected this result, and since this group had a more severe illness compared to the whole cohort of LinCoS there is a possibility that the utilized inclusion criteria and the lack of control group reduces the reliability of the conclusion that MRI abnormalities were unrelated to disease severity. However, MRI abnormalities were found both in patients who had received ICU care, as well as in patients who did not need this level of care. SWI abnormalities have been reported in the acute phase of COVID-19 infection.[5] In this cohort, SWI abnormalities were found in 8 of 35 cases, which is less frequent than reported from the Stockholm cohort published in 2020[5] where 29 of 39 patients had this finding. However, in the Stockholm cohort study, most of the patients had been on a ventilator in the ICU, whereas in the present cohort, only 54% of the patients needed ventilator care, thus indicating less severe illness in our cohort.

In the present study, there were no significant correlations between RBANS total scale score, time in hospital, or scores on the WHO Clinical Progression Scale. This finding is in contrast to previous studies which suggested that the severity of COVID-19 infection has a bearing on the degree of cognitive impairment.[10 11] In the Almeria study, early cognitive testing performed at 10 to 35 days post discharge showed a worse cognitive performance

For peer review only - http://bmjopen.bmj.com/site/about/guidelines.xhtml

among patients who required oxygen therapy during hospitalisation.[11] In the present study, where neurocognitive assessment was performed five months post discharge, no such association was found.

Results from the present study suggest that MRI findings and/or neurocognitive impairments may occur also in patients with less severe COVID-19, and future research should therefore also include evaluation of patients with COVID-19 who have either been treated in regular pandemic wards or who have not been hospitalised at all.

Strengths and limitations

The patients were carefully selected from a population-based cohort study including all confirmed cases of COVID-19 admitted to hospital during a three month period. Brain MRIs and neurocognitive assessments were analysed by an experienced neuroradiologist and two experienced neuropsychologists respectively. RBANS is considered a comprehensive test battery[36] and has been recognised as the gold standard neurocognitive battery for diagnosis and clinical trial outcome measurement in Mild Cognitive Impairment.[37] However, RBANS is a relatively brief battery and as such does not cover all aspects of cognition. The absence of premorbid cognitive assessment makes it hard to refute that any premorbid factor might have affected the results. However, when considering the background data available on the premorbid level of functioning and educational level, the poor performance on neurocognitive testing is unexpected in this cohort. In conjunction with frequent patient reports of new impairments in higher cerebral function, this point to the likelihood that these results indeed represent COVID-19-related problems.

With the lack of premorbid MRI, as well as a relatively small sample size and because it was not manageable to include a control group, it is difficult to confirm that white matter changes could be solely attributed to the COVID-19 infection. However, in cases where an MRI was performed during the acute phase of the hospitalisation, all patients subsequently developed new white matter lesions, making it plausible to assume that the COVID-19 infection contributed to these white matter lesions. This will have an impact on consideration of differential diagnoses when evaluating follow-up MRI on patients who have had COVID-19, especially in younger patients who display white matter lesions on brain MRI after a COVID-19 infection.

For peer review only - http://bmjopen.bmj.com/site/about/guidelines.xhtml

Conclusions

A majority of patients with concerning neuropsychological symptoms and/or medical history selected to undergo MRI after a clinical evaluation showed signs of possible COVID-19-related brain affection, detectable by brain MRI and/or neurocognitive test results. Deviating findings were not restricted to patients with severe disease. Thus, post-COVID-19-related changes could be considered in the differential diagnosis when white matter lesions are detected on brain MRI. Furthermore, it is important to consider post-COVID-19-related changes when dealing with patients' reports of neuropsychological deficiency, regardless of the severity of disease. As neuropsychological impairments are not always associated with abnormal brain MRI findings, a multi-professional approach would be preferable when assessing patients with persisting neuropsychological complaints following COVID-19.

References

1. Iadecola C, Anrather J, Kamel H. Effects of COVID-19 on the nervous system. *Cell* 2020
2. Romero-Sánchez CM, Díaz-Maroto I, Fernández-Díaz E, et al. Neurologic manifestations in hospitalized patients with COVID-19: The ALBACOVID registry. *Neurology* 2020;95(8):e1060-e70. doi: 10.1212/wnl.0000000000009937 [published Online First: 2020/06/03]
3. Paterson RW, Brown RL, Benjamin L, et al. The emerging spectrum of COVID-19 neurology: clinical, radiological and laboratory findings. *Brain* 2020 doi: 10.1093/brain/awaa240 [published Online First: 2020/07/09]
4. Chougar L, Shor N, Weiss N, et al. Retrospective Observational Study of Brain MRI Findings in Patients with Acute SARS-CoV-2 Infection and Neurologic Manifestations. *Radiology* 2020;297(3):E313-E23.
5. Klironomos S, Tzortzakakis A, Kits A, et al. Nervous system involvement in COVID-19: results from a retrospective consecutive neuroimaging cohort. *Radiology* 2020:202791.
6. Lu Y, Li X, Geng D, et al. Cerebral micro-structural changes in COVID-19 patients–an MRI-based 3-month follow-up study. *EClinicalMedicine* 2020;25:100484.
7. Mazza MG, Palladini M, De Lorenzo R, et al. Persistent psychopathology and neurocognitive impairment in COVID-19 survivors: effect of inflammatory biomarkers at three-month follow-up. *Brain, behavior, and immunity* 2021;94:138-47.
8. Ferrucci R, Dini M, Groppo E, et al. Long-Lasting Cognitive Abnormalities after COVID-19. *Brain sciences* 2021;11(2) doi: 10.3390/brainsci11020235 [published Online First: 2021/03/07]
9. Miskowiak K, Johnsen S, Sattler S, et al. Cognitive impairments four months after COVID-19 hospital discharge: Pattern, severity and association with illness variables. *European Neuropsychopharmacology* 2021;46:39-48.
10. Negrini F, Ferrario I, Mazziotti D, et al. Neuropsychological Features of Severe Hospitalized Coronavirus Disease 2019 Patients at Clinical Stability and Clues for Postacute Rehabilitation. *Arch Phys Med Rehabil* 2020 doi: 10.1016/j.apmr.2020.09.376 [published Online First: 2020/09/30]

For peer review only - http://bmjopen.bmj.com/site/about/guidelines.xhtml

11. Almeria M, Cejudo JC, Sotoca J, et al. Cognitive profile following COVID-19 infection: Clinical predictors leading to neuropsychological impairment. *Brain, behavior, & immunity health* 2020;9:100163. doi: 10.1016/j.bbih.2020.100163 [published Online First: 2020/10/29]
12. Raman B, Cassar MP, Tunnicliffe EM, et al. Medium-term effects of SARS-CoV-2 infection on multiple vital organs, exercise capacity, cognition, quality of life and mental health, post-hospital discharge. *EClinicalMedicine* 2021;31:100683.
13. Divanoglou A, Samuelsson K, Sjödahl R, et al. Rehabilitation needs and mortality associated with the Covid-19 pandemic: a population-based study of all hospitalised and home-healthcare individuals in a Swedish healthcare region. *medRxiv* 2021
14. von Elm E, Altman DG, Egger M, et al. The Strengthening the Reporting of Observational Studies in Epidemiology (STROBE) statement: guidelines for reporting observational studies. *The Lancet* 2007;370(9596):1453-57. doi: 10.1016/S0140-6736(07)61602-X
15. Clegg A, Young J, Iliffe S, et al. Frailty in elderly people. *The Lancet* 2013;381(9868):752-62. doi: 10.1016/S0140-6736(12)62167-9
16. Rockwood K, Song X, MacKnight C, et al. A global clinical measure of fitness and frailty in elderly people. *Cmaj* 2005;173(5):489-95.
17. Marshall JC, Murthy S, Diaz J, et al. A minimal common outcome measure set for COVID-19 clinical research. *The Lancet Infectious Diseases* 2020
18. Randolph C. RBANS update: Repeatable battery for the assessment of neuropsychological status: PsychCorp 2012.
19. Zigmond AS, Snaith RP. The hospital anxiety and depression scale. *Acta psychiatrica scandinavica* 1983;67(6):361-70.
20. Lundh Hagelin C, Wengström Y, Runesdotter S, et al. The psychometric properties of the Swedish Multidimensional Fatigue Inventory MFI-20 in four different populations. *Acta Oncologica* 2007;46(1):97-104.
21. Girard TD, Jackson JC, Pandharipande PP, et al. Delirium as a predictor of long-term cognitive impairment in survivors of critical illness. *Critical care medicine* 2010;38(7):1513.
22. Mitchell ML, Shum DH, Mihala G, et al. Long-term cognitive impairment and delirium in intensive care: A prospective cohort study. *Australian Critical Care* 2018;31(4):204-11.
23. Bjelland I, Dahl AA, Haug TT, et al. The validity of the Hospital Anxiety and Depression Scale: an updated literature review. *Journal of psychosomatic research* 2002;52(2):69-77.
24. Hinz A, Fleischer M, Brähler E, et al. Fatigue in patients with sarcoidosis, compared with the general population. *Gen Hosp Psychiatry* 2011;33(5):462-8. doi: 10.1016/j.genhosppsych.2011.05.009 [published Online First: 2011/07/14]
25. Perneger TV. What's wrong with Bonferroni adjustments. *Bmj* 1998;316(7139):1236-38.
26. Sullivan BN, Fischer T. Age-Associated Neurological Complications of COVID-19: A Systematic Review and Meta-Analysis. *Frontiers in Aging Neuroscience* 2021:374.
27. Mandal S, Barnett J, Brill SE, et al. 'Long-COVID': a cross-sectional study of persisting symptoms, biomarker and imaging abnormalities following hospitalisation for COVID-19. *Thorax* 2020 doi: 10.1136/thoraxjnl-2020-215818 [published Online First: 2020/11/12]
28. Xiong Q, Xu M, Li J, et al. Clinical sequelae of COVID-19 survivors in Wuhan, China: a single-centre longitudinal study. *Clinical Microbiology and Infection* 2020;27(1):89-95.
29. Huang C, Huang L, Wang Y, et al. 6-month consequences of COVID-19 in patients discharged from hospital: a cohort study. *The Lancet* 2021
30. Beaulieu-Bonneau S, Ouellet M-C. Fatigue in the first year after traumatic brain injury: course, relationship with injury severity, and correlates. *Neuropsychological rehabilitation* 2017;27(7):983-1001.
31. Gunning-Dixon FM, Raz N. The cognitive correlates of white matter abnormalities in normal aging: a quantitative review. *Neuropsychology* 2000;14(2):224.

For peer review only

For peer review only - http://bmjopen.bmj.com/site/about/guidelines.xhtml

32. Filley CM, Fields RD. White matter and cognition: making the connection. *Journal of neurophysiology* 2016;116(5):2093-104.
33. Kravitz DJ, Saleem KS, Baker CI, et al. A new neural framework for visuospatial processing. *Nature Reviews Neuroscience* 2011;12(4):217-30.
34. Seydell-Greenwald A, Ferrara K, Chambers CE, et al. Bilateral parietal activations for complex visual-spatial functions: Evidence from a visual-spatial construction task. *Neuropsychologia* 2017;106:194-206.
35. Agarwal S, Jain R, Dogra S, et al. Cerebral microbleeds and leukoencephalopathy in critically ill patients with COVID-19. *Stroke* 2020;51(9):2649-55.
36. Honarmand K, Lalli RS, Priestap F, et al. Natural history of cognitive impairment in critical illness survivors. A systematic review. *American journal of respiratory and critical care medicine* 2020;202(2):193-201.
37. Karantzoulis S, Novitski J, Gold M, et al. The Repeatable Battery for the Assessment of Neuropsychological Status (RBANS): utility in detection and characterization of mild cognitive impairment due to Alzheimer's disease. *Archives of clinical neuropsychology* 2013;28(8):837-44.

Contributors: LH, KS, AD, RL, UBT and IB took part in planning the study. LH, UBT and IB participated in data collection. LH, IB and KS analysed the data. LH and IB wrote the original draft of the manuscript. LH, KS, AD, RL, UBT and IB reviewed and edited the manuscript. The corresponding author attests that all listed authors meet the authorship criteria and that no others meeting the criteria have been omitted. LH is guarantor for the study.

Funding: The study was funded by the ALF grant, award/grant number N/A. The funder of the study had no role in study design, data collection, data analysis, data interpretation, or writing of the report.

Competing interests: None declared.

Ethical approval: The study has been approved by the Swedish Ethical Review Authority (Dnr 2020-03029 and 2020-04443).

For peer review only - http://bmjopen.bmj.com/site/about/guidelines.xhtml

Data sharing: The Public Access to Information and Secrecy Act in Sweden prohibits us from making individual level data publicly available, thus no additional data will be made available.

Transparency: The lead author affirms that this manuscript is an honest, accurate, and transparent account of the study being reported; that no important aspects of the study have been omitted; and that any discrepancies from the study as planned have been explained.

Patient and public involvement: Formally, due to the early stage of the COVID-19 pandemic in Sweden, neither patients nor the public were involved in the design or planning of this study. We intend to seek patient and public involvement in the development of an appropriate method of dissemination. Results from the study will be presented to the public through public service. The patient organisation for COVID-19 in Sweden will be contacted and the results from the study will be shared. In addition, the results will be presented at relevant medical conferences.

Provenance and peer review: Not commissioned; externally peer reviewed.

Acknowledgements: We thank Lars Valter at Forum Östergötland, Faculty of Medicine and Health Sciences, Linköping University, Linköping, Sweden, for statistical advice; and Ninnie Håkansson at the Radiology Department of Linköping University hospital for invaluable assistance with MRI bookings. Last, a special acknowledgement to Agnes Andersson, Julia Eriksson, Jacob Lennartsson, Malin Lindh and Mollie Rönn Holmström for valuable assistance with performing the neurocognitive evaluations.

Figure legends:

Figure 1 Flowchart of the inclusion and exclusion process of patients previously hospitalised with COVID-19

For peer review only - http://bmjopen.bmj.com/site/about/guidelines.xhtml

Figure 2 T2FLAIR images for one of the patients. A and C, superior row, are from the MRI examination performed during the acute phase. B and D, inferior row, are from the MRI examination at late follow-up. New white matter lesions appear at the follow-up MRI, with a typical distribution with a predilection for the parietal and frontal lobes

Figure 3 Susceptibility weighted image (SWI) for one of the patients at follow-up MRI. There are multiple SWI abnormalities, mainly located in the grey-white matter junction

For peer review only

For peer review only - http://bmjopen.bmj.com/site/about/guidelines.xhtml

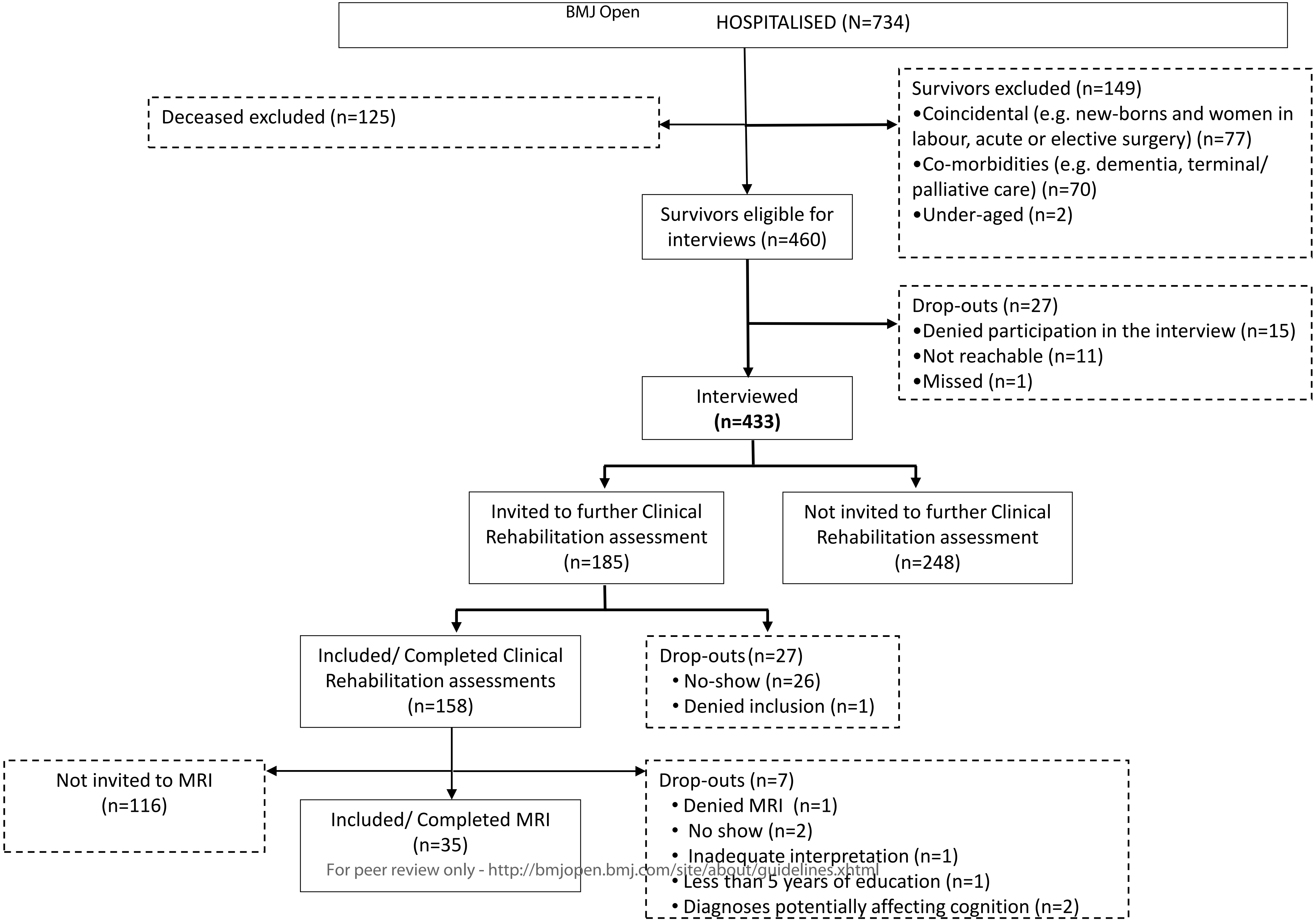
HOSPITALISED (N=734)
Deceased excluded (n=125)
Survivors excluded (n=149)
•Coincidental (e.g. new-borns and women in labour, acute or elective surgery) (n=77)
•Co-morbidities (e.g. dementia, terminal/ palliative care) (n=70)
•Under-aged (n=2)
Survivors eligible for interviews (n=460)
Drop-outs (n=27)
•Denied participation in the interview (n=15)
•Not reachable (n=11)
•Missed (n=1)
Interviewed (n=433)
Invited to further Clinical Rehabilitation assessment (n=185)
Not invited to further Clinical Rehabilitation assessment (n=248)
Included/ Completed Clinical Rehabilitation assessments (n=158)
Drop-outs (n=27)
• No-show (n=26)
• Denied inclusion (n=1)
Not invited to MRI (n=116)
Included/ Completed MRI (n=35)
Drop-outs (n=7)
• Denied MRI (n=1)
• No show (n=2)
• Inadequate interpretation (n=1)
• Less than 5 years of education (n=1)
• Diagnoses potentially affecting cognition (n=2)

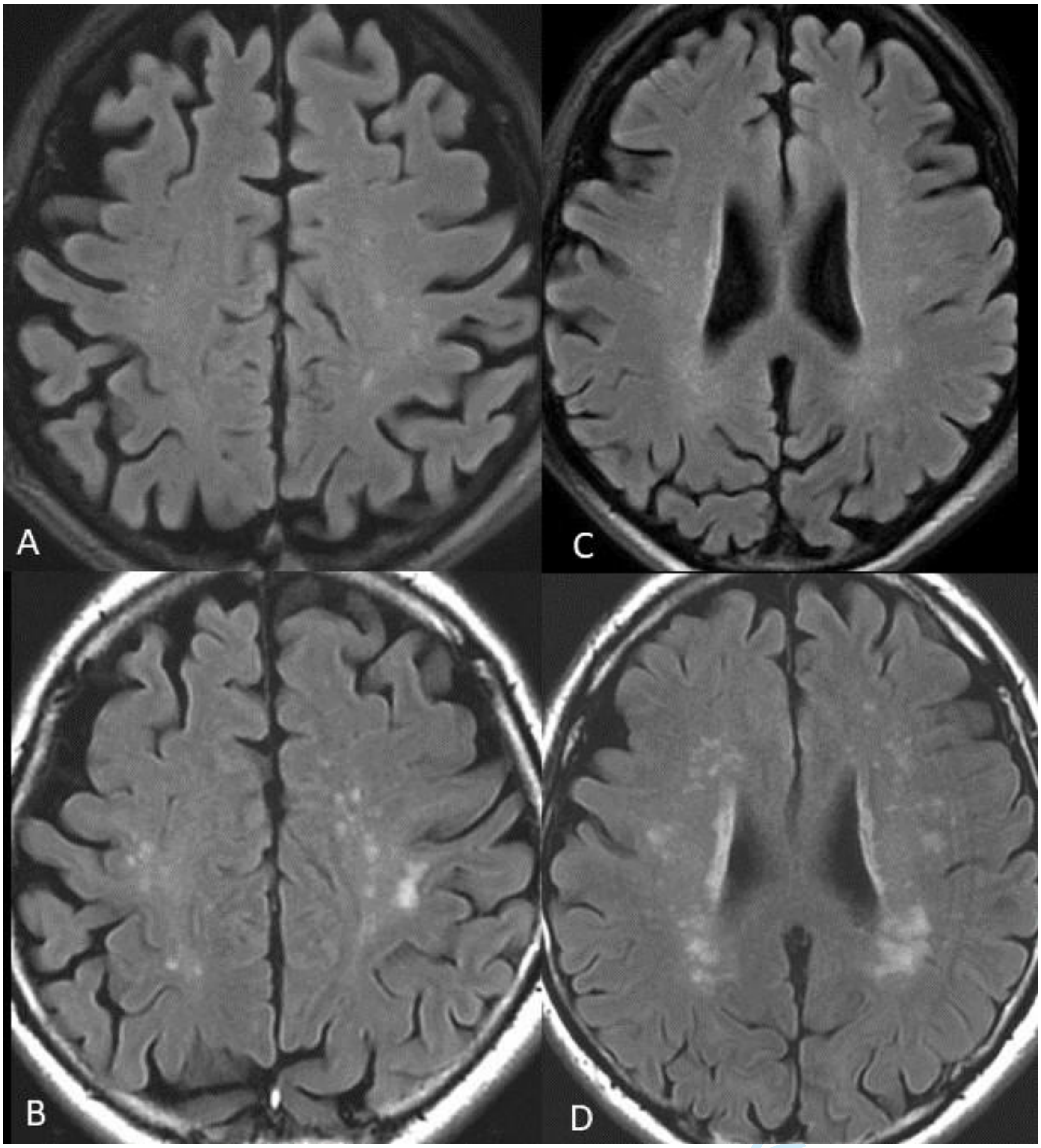

Fig 2 T2FLAIR images for one of the patients. A and C are from the MRI examination performed during the acute phase. B and D are from the MRI examination at late follow-up. New white matter lesions appear at the follow-up MRI, with a typical distribution with a predilection for the parietal and frontal lobes

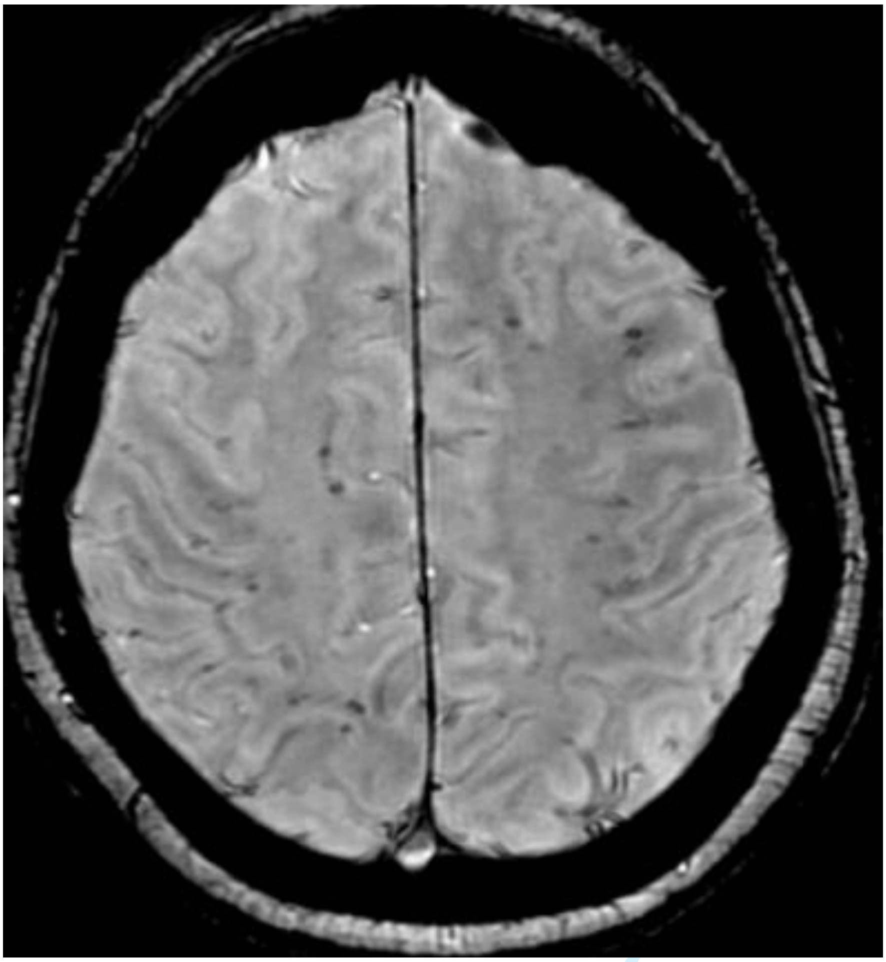

Fig 3 Susceptibility weighted image (SWI) for one of the patients at follow-up MRI. There are multiple SWI abnormalities, mainly located in the grey-white matter junction

view only

Supplemental Table 1. Characteristics for all included patients hospitalised in a Swedish geographical region during the period March 1 to May 31, 2020 with a laboratory confirmed COVID-19; those eligible for interview; those invited for a clinical assessment and those who underwent an MRI

	**All hospitalised (n=734)**	**Eligible for interview (n=460)**	**Invited to a clinical assessment (n=185)**	**MRI performed (n=35)**
Age, median (IQR) years	67 (52-79)	61 (49-74)	59 (49-67)	59 (51-66)
Men/women, n (%)	388 /346 (53/47)	257/203 (56/44)	104/81 (56/44)	28/7 (80/20)
Days in hospital, median (IQR)	6 (3-12)	6 (3-13)	7 (3-19)	18 (7-47)
ICU care, need/no need, n (%)	102/632 (14/86)	72/388 (16/84)	49/136	20/15 (57/43)
Premorbid function category 1/2/3/4/missing, n (%)	210/188/226/100/10 (29/26/31/14/1)	183/142/125/9/1 (40/31/27/2/0)	92/58/32/2/1 (51/31/17/1/1)	20/12/3/0/0 (57/34/9/0/0)
WHO Clinical Progression Scale 4/5/6/7/8/9/10/missing, n (%)	230/261/47/0/25/34/130/7 (31/36/6/0/3/5/18/1)	139/221/41/0/25/34/0/0 (30/48/9/0/5/7/0/0)	51/71/18/0/20/25/0/0 (28/38/10/0/11/14/0/0)	4/8/4/0/6/13/0/0 (11/23/11/0/17/37/0/0)

Categories of premorbid function: 1. No or mild frailty, no restriction in daily life; 2. Moderate frailty, mobile and independent, but unable to handle physically demanding activities or work; 3. Considerable frailty, ability to perform activities of daily living, but in periods confined to bed or chair; and 4. Severe frailty, not able to perform activities of daily living and/or confined to bed or chair. Dementia necessitating care. WHO Clinical Progression Scale: 4. Hospitalised, moderate disease, no oxygen therapy; 5. Hospitalised, moderate disease, oxygen by mask or nasal prongs; 6. Hospitalised, severe diseases, oxygen by non-invasive ventilation or high flow; 7. Hospitalised, severe diseases, intubation and mechanical ventilation, $pO_2/FiO_2 \geq 150$ or $SpO_2/FiO_2 \geq 200$; 8. Hospitalised, severe diseases, mechanical ventilation pO_2/FiO_2 <150 (SpO_2/FiO_2 <200) or vasopressors; and 9. Hospitalised, severe diseases, mechanical ventilation pO_2/FiO_2 <150 and vasopressors, dialysis, or ECMO. Number of symptoms affecting daily life as reported by patients 4 months post discharge.

For peer review only

Supplemental table 2 MRI findings in patients previously hospitalized with COVID-19

Gender	Age (years in intervalls)	Previous MRI in acute phase	SWI abnormalities on MRI	White matter lesions on MRI	Confluent white matter lesions	New white matter lesions	Normal MRI
F	40-49						X
M	40-49						X
M	50-59			X			
F	60-69			X			
M	50-59						X
M	50-59			X	X		
M	40-49						X
M	60-69			X			
M	70-79			X			
F	60-69			X			
M	70-79		X		X		
F	50-59						X
M	60-69	X	X	X	X	X	
M	50-59						X
M	50-59			X			
F	50-59			X			
F	60-69		X				
M	60-69			X	X		
M	50-59			X	X		
M	40-49			X			
M	40-49						X
M	50-59						X
F	60-69						X
M	60-69			X			
M	70-79	X	X	X		X	
M	50-59			X			
M	70-79	X		X	X	X	
M	40-49			X			
M	60-69	X	X	X		X	
M	50-59						X
M	60-69			X			
M	50-59	X		X		X	
M	80-89		X		X		
M	60-69	X	X	X		X	
M	50-59		X				

For peer review only

Supplemental table 3 Background data and data from the clinical assessment on patients who did/did not complete all RBANS subtests

	Patients who completed all RBANS subtests	Patients who did not complete all RBANS subtests	P [a)]	Patients who completed all RBANS subtests (n)	Patients who did not complete all RBANS subtests (n)	P[b)]	Patients who completed all RBANS subtests (n)	Patients who did not complete all RBANS subtests (n)
Age, median (IQR)[a]	59 (51-66)	62.5 (51.25-76)	n.s.				31	4
Men/women, n[b]				24/7	4/0	n.s.	31	4
Days in hospital, median (IQR)[a]	18 (8-47)	13 (4.75-86.5)	n.s.				31	4
ICU care, need/no need, n[b]				18/13	2/2	n.s.	31	4
Mechanical ventilation, need/no need, n[b]				17/14	2/2	n.s.	31	4
Premorbid function category 1/2/3/4, n[b]				19/10/2/0	1/2/1/0	n.s.	31	4
WHO Clinical Progression Scale 4/5/6/7/8/9, n[b]				3/7/4/0/6/11	1/1/0/0/0/2	n.s.	31	4
Educational level 1/2/3, n[b]				7/15/9	3/0/4	n.s.	31	4
Self-estimated previous health, n[b]				13/9/8/0/0	0/3/1/0/0	n.s.	30	4
Self-estimated present health, n[b]				4/4/16/6/0	0/0/2/2/0	n.s.	30	4
Distribution of impaired cognitive results, category 1/2/3, n[b]				8/6/17	2/0/2	n.s.	31	4
MFI, above cut-off/not above cut-off, n[b]				23/7	3/1	n.s.	30	4
HADS-depression above/not above cut-off, n[b]				3/28	2/2	n.s.	30	3
HADS-anxiety above/not above cut-off, n[b]				4/27	2/2	n.s.	30	3
MRI findings abnormal/normal, n[b]				22/9	3/1	n.s.	31	4

a) Median test b) Fisher test for comparison of frequencies. The difference was considered significant at $P<0.05$. n.s.=non-significant. n=number of patients. Categories of premorbid function: 1. No or mild frailty, no restriction in daily life; 2. Moderate frailty, mobile and independent, but unable to handle physically demanding activities or work; 3. Considerable frailty, ability to perform activities of daily living, but with periods confined to bed or chair; and 4. Severe frailty, not able to perform activities of daily living and/or confined to bed or chair. Dementia necessitating care. WHO Clinical Progression Scale: 4. Hospitalised, moderate disease, no oxygen therapy; 5. Hospitalised, moderate disease, oxygen by mask or nasal prongs; 6. Hospitalised, severe diseases, oxygen by non-invasive ventilation or high flow; 7. Hospitalised, severe diseases, intubation and mechanical ventilation, $pO_2/FiO_2 \geq 150$ or $SpO_2/FiO_2 \geq 200$; 8. Hospitalised, severe diseases, mechanical ventilation $pO_2/FiO_2 <150$ ($SpO_2/FiO_2 <200$) or vasopressors; and 9. Hospitalised, severe diseases, mechanical ventilation $pO_2/FiO_2 <150$ and vasopressors, dialysis, or extracorporeal membrane oxygenation. Categories of educational level: 1, up to 9 years of school; 2, 9 to 12 years of school; and 3, more than 12 years of school. Categories of self-estimated health (previous and present): 1, Excellent; 2, Very good; 3, Good; 4, Fair; and 5, Poor. RBANS=The Repeatable Battery for Assessment of Neuropsychological Status Categories of impaired cognitive results: 1, scores 2SD below mean according to norms in at least two RBANS indexes, or 1.5SD below the mean according to the norms on at least three RBANS indexes, was considered severe impairment; 2, scores 2SD below the mean according to the norms on one of the RBANS indexes or 1.5SD below the mean according to the norms on two RBANS indexes, was considered mild/moderate impairment; 3, scores not included in categories 1 and 2, considered not mild/moderate/severe impairment. RBANS cut-off (scores at least 2SDs below age-adjusted population mean of 100, SD 15 according to norms). MFI=Multidimensional Fatigue Inventory. MFI cut-off for clinically significant fatigue ≥53. HADS= Hospital Anxiety and Depression Scale. HADS cut-off for potential depression/anxiety ≥8.

For peer review only

STROBE Statement—Checklist of items that should be included in reports of ***cohort studies***

	Item No	Recommendation	Page No
**Title and abstract**	1	(*a*) Indicate the study's design with a commonly used term in the title or the abstract	2
		(*b*) Provide in the abstract an informative and balanced summary of what was done and what was found	2
**Introduction**			
Background/rationale	2	Explain the scientific background and rationale for the investigation being reported	3
Objectives	3	State specific objectives, including any prespecified hypotheses	3
**Methods**			
Study design	4	Present key elements of study design early in the paper	3-4
Setting	5	Describe the setting, locations, and relevant dates, including periods of recruitment, exposure, follow-up, and data collection	3-4
Participants	6	(*a*) Give the eligibility criteria, and the sources and methods of selection of participants. Describe methods of follow-up	3-4
		(*b*) For matched studies, give matching criteria and number of exposed and unexposed	
Variables	7	Clearly define all outcomes, exposures, predictors, potential confounders, and effect modifiers. Give diagnostic criteria, if applicable	4-7
Data sources/ measurement	8*	For each variable of interest, give sources of data and details of methods of assessment (measurement). Describe comparability of assessment methods if there is more than one group	4-7
Bias	9	Describe any efforts to address potential sources of bias	6, 8
Study size	10	Explain how the study size was arrived at	4
Quantitative variables	11	Explain how quantitative variables were handled in the analyses. If applicable, describe which groupings were chosen and why	7-8
Statistical methods	12	(*a*) Describe all statistical methods, including those used to control for confounding	7-8
		(*b*) Describe any methods used to examine subgroups and interactions	7-8
		(*c*) Explain how missing data were addressed	8
		(*d*) If applicable, explain how loss to follow-up was addressed	n.a.
		(*e*) Describe any sensitivity analyses	
**Results**			
Participants	13*	(a) Report numbers of individuals at each stage of study—eg numbers potentially eligible, examined for eligibility, confirmed eligible, included in the study, completing follow-up, and analysed	Fig1
		(b) Give reasons for non-participation at each stage	Fig1
		(c) Consider use of a flow diagram	Fig1
Descriptive data	14*	(a) Give characteristics of study participants (eg demographic, clinical, social) and information on exposures and potential confounders	Table 1
		(b) Indicate number of participants with missing data for each variable of interest	In each table
		(c) Summarise follow-up time (eg, average and total amount)	6
Outcome data	15*	Report numbers of outcome events or summary measures over time	6-7

For peer review only - http://bmjopen.bmj.com/site/about/guidelines.xhtml

For peer review only

Main results	16	(*a*) Give unadjusted estimates and, if applicable, confounder-adjusted estimates and their precision (eg, 95% confidence interval). Make clear which confounders were adjusted for and why they were included<br>(*b*) Report category boundaries when continuous variables were categorized<br>(*c*) If relevant, consider translating estimates of relative risk into absolute risk for a meaningful time period	8-9
Other analyses	17	Report other analyses done—eg analyses of subgroups and interactions, and sensitivity analyses	8-9
**Discussion**			
Key results	18	Summarise key results with reference to study objectives	9-11
Limitations	19	Discuss limitations of the study, taking into account sources of potential bias or imprecision. Discuss both direction and magnitude of any potential bias	11-12
Interpretation	20	Give a cautious overall interpretation of results considering objectives, limitations, multiplicity of analyses, results from similar studies, and other relevant evidence	9-11
Generalisability	21	Discuss the generalisability (external validity) of the study results	9-11
**Other information**			
Funding	22	Give the source of funding and the role of the funders for the present study and, if applicable, for the original study on which the present article is based	15

*Give information separately for exposed and unexposed groups.

Note: An Explanation and Elaboration article discusses each checklist item and gives methodological background and published examples of transparent reporting. The STROBE checklist is best used in conjunction with this article (freely available on the Web sites of PLoS Medicine at http://www.plosmedicine.org/, Annals of Internal Medicine at http://www.annals.org/, and Epidemiology at http://www.epidem.com/). Information on the STROBE Initiative is available at http://www.strobe-statement.org.

For peer review only
